# Stimulus-responsive light-harvesting complexes based on the pillararene-induced co-assembly of β-carotene and chlorophyll

**DOI:** 10.1038/ncomms12042

**Published:** 2016-06-27

**Authors:** Yan Sun, Fang Guo, Tongfei Zuo, Jingjing Hua, Guowang Diao

**Affiliations:** 1 College of Chemistry and Chemical Engineering, Yangzhou University, Yangzhou, Jiangsu 225002, China

## Abstract

The locations and arrangements of carotenoids at the subcellular level are responsible for their designated functions, which reinforces the necessity of developing methods for constructing carotenoid-based suprastructures beyond the molecular level. Because carotenoids lack the binding sites necessary for controlled interactions, functional structures based on carotenoids are not easily obtained. Here, we show that carotene-based suprastructures were formed via the induction of pillararene through a phase-transfer-mediated host–guest interaction. More importantly, similar to the main component in natural photosynthesis, complexes could be synthesized after chlorophyll was introduced into the carotene-based suprastructure assembly process. Remarkably, compared with molecular carotene or chlorophyll, this synthesized suprastructure exhibits some photocatalytic activity when exposed to light, which can be exploited for photocatalytic reaction studies of energy capture and solar conversion in living organisms.

## Abstract (web summary)

Carotenoids are photoactive organic pigments found in many plants. Here, the authors report the self-assembly of carotenoid suprastructures by forming a pillararene host-guest complex, and show that these structures display functionalities including stimuli responsiveness and photocatalytic activity.

## Graphical abstract

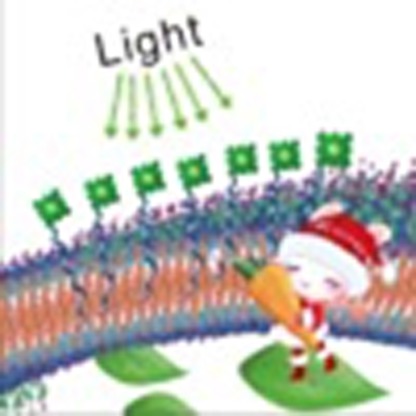

Carotenoids are a diverse group of structurally related compounds^1^ that perform various functions in living organisms^2^. Their conjugated double-bond systems determine their photochemical properties and their chemical reactivities^3^, which, in turn, influence the properties of these subcellular structures^4^. They are relevant to human and animal processes related to nutrition^5^, immune systems^6^ and antioxidation^7^. Their roles in plants and microorganisms are ascribed mostly to acting as a stabilizing structure for light-harvesting complexes (LHCs)^8^, photoprotection^9^, absorbing light and transferring energy to chlorophyll pigments^10^, which acts as a driving force for photosynthesis^11^. Furthermore, the molecular geometry and specific interactions with other molecules in the structures are critical for ensuring that the carotenoids fit into the suprastructures in the correct location and orientation^12^. Considerable effort has been devoted to investigating the biochemistry and molecular biology of carotenoids^13^. Nevertheless, in the fields of both nutrition utilization and suprastructure construction, suprastructure development has been limited by the hydrophobic nature of carotenoids^14^.

Recently, in the carotenoid-related fields, this problem has been partially solved through the use of hydrophilic vectors^15^. For example, natural carotene has been wrapped into hydrophilic vectors by spinning disk processing to construct a carotene-loaded particle^16^. Notably, supramolecular studies have been focused primarily on the characterization of the natural photosynthesis machinary^17^. The design and synthesis of programmable entities based on carotenoids still faces many issues due to the low solubility of natural pigments in aqueous media. Thus, no carotenoid molecules have been introduced as building blocks to form suprastructures that mimic natural structures. To solve these problems, synthetic water-soluble analogues have often been used instead of natural pigments as building units^18^. Compared with the successful fabrication of many synthetic supramolecular systems based on hydrophilic biomolecules, including peptides^19^, fatty acids^20^, nucleotides^21^ and porphyrins^22^, the preparation of synthetic suprastructures based on hydrophobic biomolecules remains in the initial stages. Therefore, the development of effective strategies for assembling carotenoids into intricate and customizable large-scale systems that perform the same physiological functions as they perform *in vivo* is now essential^23^.

Fortunately, supramolecular chemistry provides a new framework to tackle this difficult question. Thus far, supramolecular macrocycles, such as crown ethers^24^,^25^,^26^, cyclodextrins^27^ and calixarenes^28^,^29^, have been used for various applications. Pillararenes^30^,^31^, which are composed of hydroquinone units linked by methylene bridges at the para positions, are an especially interesting new class of macrocyclic host in supramolecular chemistry. Their unique, intrinsically rigid and symmetrical pillar architecture endows them with an outstanding ability to selectively bind various types of guest molecules^32^. This property has been used to construct various interesting supramolecular systems^33^, including nanomaterials^34^, sensors^35^, ion channels^36^ and drug-delivery systems^37^.

In this article, β*-*carotene (β-CAR) was selected as a type of carotenoid to represent the carotenoid family. A synthetic water-soluble carboxyl-modified pillararene (WP5) was also chosen because its cavity dimensions are appropriate for the accommodation of β-CAR, which could further induce the construction of a β-CAR-based substructure. We envisioned that β-CAR might bind WP5 in water to achieve WP5⊃β-CAR complexation (WCC) via the hydrophobic effect. Then, the resulting amphiphilic WCC might be able to form a β-CAR-containing suprastructure in water via self-assembly. On the basis of the fascinating properties exhibited by natural chlorophyll/carotenoid complexes in photosynthesis, chlorophyll-b (Chl-b) was selected as the co-assembly factor participating in the preparation process with WCC, and a similar suprastructure of Chl-b-containing LHC was synthesized. These hydrophilic complexes possess a suite of unusual properties, including spontaneous growth, fusion, pH stimulus responsiveness and even some photocatalytic activity.

## Results

### Host–guest interaction between WP5 and β-CAR

**Figure 1:**
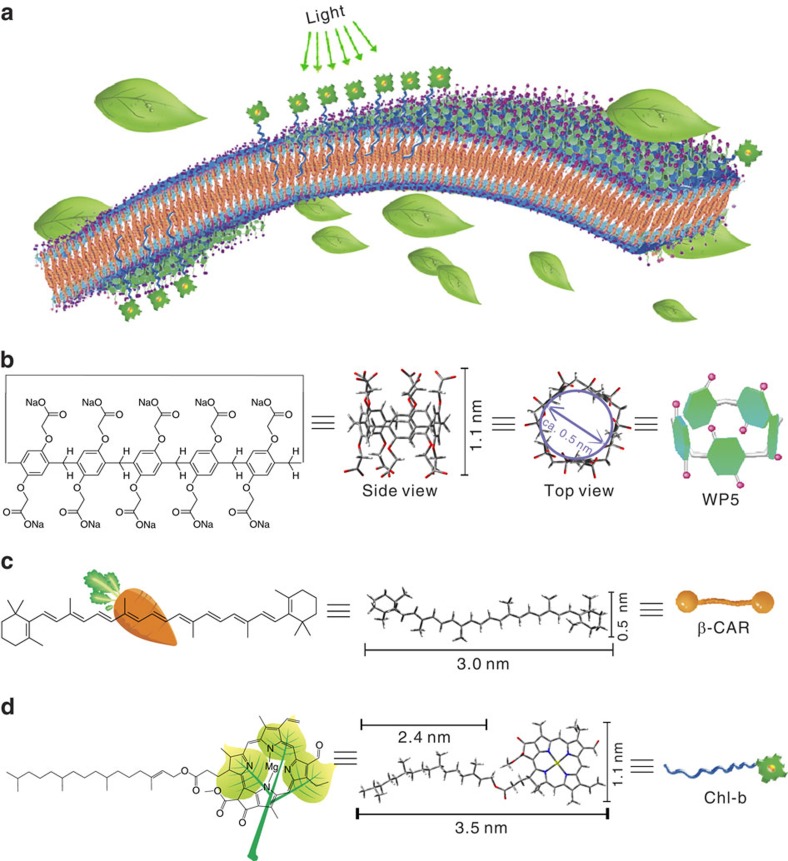
Structural model of building blocks. (**a**) LHC containing Chl-b formed by (**b**) WP5, (**c**) β-CAR and (**d**) Chl-b. For clarity, the β-CAR-based hydrophobic interior layer is orange, and the WP5-based hydrophilic exterior layer is green.

**Figure 2:**
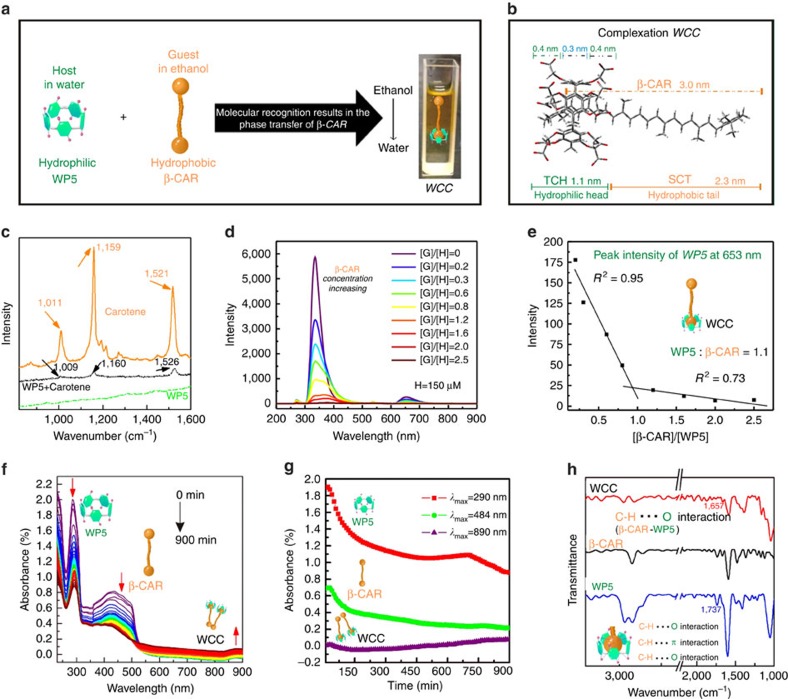
Synthesis of the WCC. (**a**) A schematic displaying the synthetic route to the WCC. (**b**) Simulated structure of the WCC. (**c**) Raman spectra of β-CAR and the WCC. (**d**) Fluorescence spectra of WP5 (150 μM) on addition of β-CAR in ethanol/water (excited at 268 nm) at room temperature. (**e**) The fluorescence intensity changes of WP5 at 653 nm. (**f**) Time-dependent ultraviolet–visible spectra showing the formation of the WCC, as indicated by the changes in the intensities of the peaks at 290 and 484 nm (20 min each). (**g**) Plot of absorbance intensity as a function of time (20 min each). (**h**) FT-IR spectra of WP5, β-CAR and the WCC.

The pillar[5]arene carboxyl derivative WP5 was prepared according to published procedures^38^ and was identified by ^1^H-NMR spectroscopy after being dissolved in D_2_O. According to the results of Gaussian 09 calculations, the internal diameter of the cavity is 0.5 nm (ref. 32 and the length of the WP5 is 1.1 nm. Within the as-prepared synthetic LHC systems, the role of WP5 (Fig. 1b) is primarily to improve the solubility of β-CAR in aqueous environments and to further induce the hierarchical arrangement of β-CAR during assembly. β-CARs are naturally abundant pigments that are extremely hydrophobic and have low solubility in water. As shown in the energy-minimized structure of β-CAR (Fig. 1c), the length and width of β-CAR are ∼3.0 and 0.5 nm, respectively. A remarkable feature of β-CAR is that its molecular shape and size (a slight twist of conformation) fit well within the cavity of WP5, which provides the molecular basis for the interaction between WP5 and β-CAR. β-CAR units serve as the structural skeleton, providing photoprotective functions and binding sites for Chl-b (Fig. 1d). Chl-b molecules serve as the light-absorbing and energy-transferring components; they can attach to the hydrophobic domains by inserting their long alkyl chains into WCC. Given that the porphyrin head group of Chl-b exhibits photocatalytic activity, it is a rational choice for a functional component for integrating the photoprotective properties of β-CAR. The length of the hydrophobic tail of Chl-b (2.4 nm) matches the length of the hydrophobic moieties of WCC (2.3 nm) well. Thus, the hydrophobic tail of Chl-b could be used to anchor Chl-b to the hydrophobic segments of WCC and further facilitate the investigation of photosynthesis on the supramolecular level. In addition, WP5 as the carboxylic sodium salt and its precipitation from water as the corresponding carboxylic acid could be reversibly adjusted by changing the pH of the solution, which could endow the proposed suprastructure with pH responsiveness. The samples were prepared by a simple procedure. β-CAR powder was first dissolved in ethanol, and then, 2 ml of this β-CAR solution (150 μM) was added to 2 ml of a dilute sodium hydroxide aqueous solution of WP5 (150 μM) using a pipette, without stirring. The formation of WCC (Fig. 2a) is likely to be mainly driven by hydrophobic interactions between the hydrophobic cavity of WP5 and the lipophilic β-CAR. As shown in Fig. 2b, a ‘tadpole-like' WCC with the bulky group at one side of β-CAR was threaded into the cavity of WP5, and the residue of β-CAR protruding from the WP5 cavity. The TCH (ten-carboxylic acid head) segment is the hydrophilic region with an extended length of 1.1 nm, and the SCT (single-carotene tail) is the segment (2.3 nm) that possesses a long hydrophobic tail that imparts amphiphilic character to WCC. The complexation between WP5 and β-CAR was first demonstrated by ^1^H-NMR spectroscopy. As shown in Supplementary Figs 1, 2, the proton peaks H_3_ of the methylene moieties at both rims were split into quartets from singlets, likely because of the inclusion of terpene in the cavity of WP5 restricted the swinging of the constituent units, which led to the loss of internal symmetry of H_3_ protons. At the same time, downfield shifts of the aromatic protons H_1_ and H_2_ of WP5 were observed. In contrast, remarkable upfield chemical shifts of the methylene protons (H_b_, H_c_ and H_d_) and methyl protons (H_a_ and H_e_) of the bulky terpene were observed (Δδ(H_b_)=0.228 p.p.m., Δδ(H_c_)=0.259 p.p.m., Δδ(H_d_)=0.354 p.p.m., Δδ(H_a_)=0.160 p.p.m. and Δδ(H_e_)=0.150p.p.m.) on the addition of WP5 due to the shielding effect of the electron-rich cavities of WP5 toward β-CAR, which clearly demonstrated the inclusion of the terpene section of β-CAR into the hydrophobic WP5 cavity. Moreover, the signals derived from protons H_g_ (Δδ(H_g_)=−0.027 p.p.m.) and methyl protons (H_f_) on linear unsaturated carbon chain shifted from 6.1–6.8 p.p.m. to downfield 6.6–6.3 p.p.m. (Δδ(H_f_)=−0.5 p.p.m.). A similar phenomenon was also observed for the inclusion complexation between water-soluble pillar[5]arene and guest in previous reports^39^,^40^. The above results revealed that the cavity was fully threaded by β-CAR with the protons H_a_, H_b_, H_c_, H_d_ and H_e_ in the hydrophobic WP5 cavity and that the other protons on a linear unsaturated carbon chain (H_f_, and H_g_) protruded out of the cavity. The binding affinity for such host–guest inclusion might be mainly driven by the hydrophobic interactions. The generation of β-CAR-based WCC was then supported by Raman spectroscopy (Fig. 2c). The features of WCC in the 1,600–900 cm^−1^ region are similar to those of the pure β-CAR spectra^41^: the peak at 1,526 cm^−1^ originates from the stretching modes of the conjugated C=C bonds; the peak at 1,160 cm^−1^ arises from a mixture of C=C and C–C bond stretching modes with C–H bending modes; and the peak at 1,009 cm^−1^ is attributed to the stretching modes of C–CH_3_ bonds between the main chain and the side methyl carbons. These peaks indicate that β-CAR is present in the WCC. In addition, the ν_1_ band assigned to C=C stretching (which is sensitive to host–guest interactions) blue-shifted (5 cm^−1^) from 1,521 to 1,526 cm^−1^, which not only provided further direct evidence of host–guest interactions between WP5 and β-CAR, but also further confirmed that the bulky groups at the ends of the β-CAR units were inserted into the cavity^42^.

The encapsulation of a guest (G) in the hydrophobic cavity might also influence the polarizability of the host (H) cavity^43^, which can be characterized by the fluorescence spectra. As shown in Fig. 2d, different (G)/(H) mole ratios ((H)=150 μM) demonstrated that the confinement of β-CAR in the cavity strongly affects the fluorescence behaviour. The stoichiometry of the WCC between WP5 and β-CAR was further investigated based on the fluorescence spectra. The peak at 653 nm was selected for the investigation, which revealed a 1:1 binding stoichiometry between WP5 and β-CAR (Fig. 2e). Notably, the peak at 654 nm was selected because of the emission peak overlap of WP5 and β-CAR at 334 nm (Supplementary Fig. 3a–f). Furthermore, the fluorescence spectra of (G)/(H) at a higher host concentration ((H)=200 μM) were also monitored, as also shown in (Supplementary Fig. 3g–i). The peak at 375 nm was selected for the investigation of the intensity change of β-CAR (this peak could not be observed when the WP5 concentration was 150 μM, which resulted in a relatively low concentration of β-CAR), and the peak at 653 nm was selected for the investigation of the intensity change of WP5. The results of both of these experiments further demonstrated that the (H)/(G)-binding stoichiometry is 1:1 (ref. 34). According to the fluorescence intensity change in Fig. 2d, the association constant (*K*_a_) of WP5**⊃**β-CAR was calculated to be (2.34±2.06) × 10^5^ M^−1^ using a non-linear curve-fitting method (Supplementary Fig. 3j). Aside from the above fluorescence results, the stoichiometry of complexation for WP5⊃β-CAR was further proven by Job's plot method using ultraviolet–visible spectroscopy, which confirmed the 1:1 binding stoichiometry for WP5⊃β-CAR complexation (Supplementary Fig. 4)^44^. To gain further insight into the dynamic interaction between WP5 and β-CAR, we acquired 45 sequential ultraviolet–visible spectra at 20-min intervals for the same sample. As shown in Fig. 2f, the intensities of the characteristic peaks at ∼290 nm and 484 nm (attributed to the absorbance of WP5 and β-CAR, respectively) decreased from 0 to 900 min. Meanwhile, the emergence and continued increase in intensity of a peak at 890 nm were observed, providing another piece of evidence for the formation of the WCC. Figure 2g clearly shows that the increase in intensity of the peak at 890 nm is associated with a concomitant decrease in the intensities of the features at 290 nm and 484 nm, which might be caused by the formation of microaggregates (Supplementary Fig. 5) and leads to the decrease of WP5 and β-CAR absorption intensity. Further evidence for the existence of the WCC was obtained by Fourier-transform infrared (FT-IR) spectroscopy, which shows that hydrogen bonds formed in the WCC (between the –COO^–^ of WP5 and the CH_3_– of β-CAR). As shown in Fig. 2h, in the spectrum of the WP5 before self-assembly, the absorbance at 1,737 cm^−1^ is attributed to a non-hydrogen-bonded –COOH group, which implies the presence of free carboxyl groups^45^. In contrast, this stretching vibration at 1,737 cm^−1^ disappeared after the complexation. These results show that strong and orderly hydrogen bonds were formed in the WCC.

### Construction of HMS based on the WCCs

**Figure 3:**
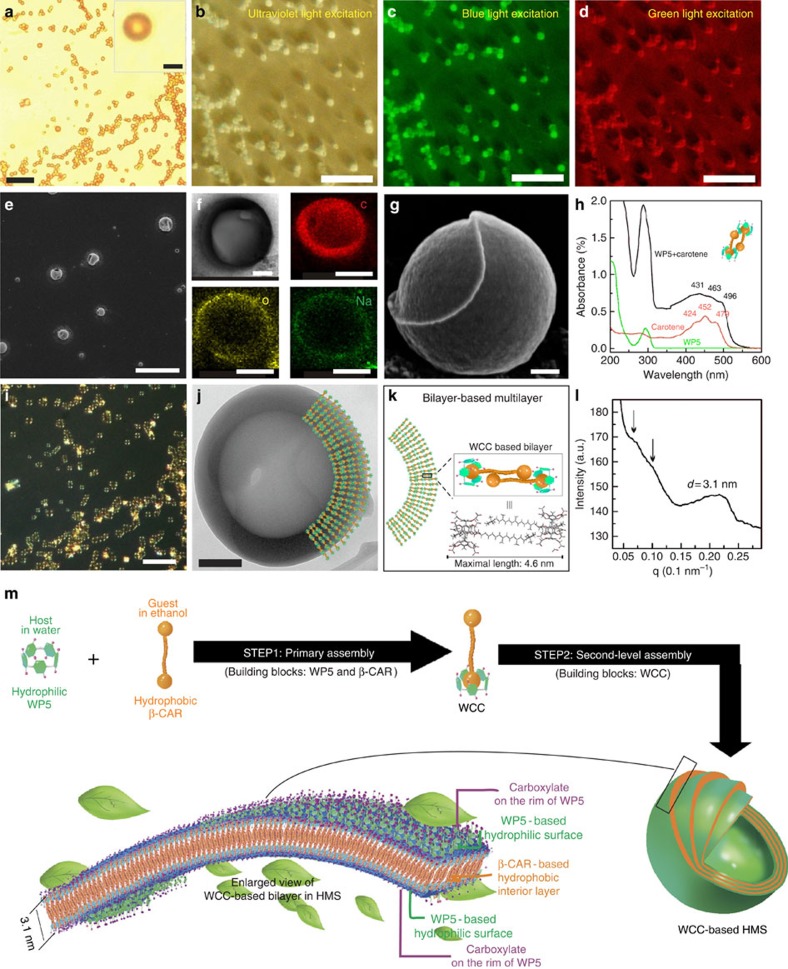
Synthesis of WCC-based HMS. (**a**) OM image of HMSs over a large area. Scale bar, 10 μm (inset: enlarged image of an HMS; Scale bar, 1 μm). Large-area fluorescence microscopy images of HMSs under (**b**) Ultraviolet-light excitation, (**c**) blue-light excitation and (**d**) green-light excitation. (**e**) SEM image of HMSs. Scale bar, 10 μm. (**f**) Top left, TEM image of an enlarged WCC-based HMS; Scale bar, 500 nm. EDX mapping images of a WCC-based HMS: top right, distribution of element C; lower left, distribution of element O; lower right, distribution of element Na. Scale bar, 1 μm. (**g**) SEM image of an HMS with a broken shell. Scale bar, 200 nm. (**h**) ultraviolet–visible spectra of HMSs, β-CAR and WP5. (**i**) Polarized image of HMSs (the colour in this image is false). Scale bar, 10 μm. (**j**) TEM image of an HMS. Scale bar, 200 nm. (**k**) Cartoon of WCC-based multilayers in the HMSs. (**l**) SAXS profile of the HMS sample (the final concentration of both HMS and β-CAR is 75 μM) with an acquisition time of 5 h. (**m**) Schematic of the HMS formation process.

WCCs were then utilized as building blocks in the construction of light-harvesting antenna complexes (LHCs). After the as-prepared solutions were aged for 7 days, orange aggregates appeared in the solutions. As shown in Fig. 3a (Supplementary Fig. 6), the aggregates were determined to be hollow microspheres (HMSs) based on optical microscopy (OM) observations, and the orange colour of the wall confirmed the homogeneous distribution of β-CAR across the HMSs. The DLS result showed that the WP5**⊃**β-CAR-based HMS had an average diameter of 1,855 nm (Supplementary Fig. 5b). Autofluorescence is exhibited in Fig. 3b–d (Supplementary Fig. 7) and is attributed to the presence of the conjugated system in β-CAR and WP5. Scanning electron microscopy (SEM) micrographs further confirmed the microspherical morphology of the HMSs (Fig. 3e, Supplementary Fig. 8). The elemental mapping analysis demonstrated the homogeneous distribution of O and Na, which are characteristic elements in WP5 (Fig. 3f), across the HMSs, providing powerful evidence that the HMSs are composed of WP5. Interestingly, an HMS with a partially broken shell was observed (Fig. 3g) by SEM. The layer peeled off in the form of an arc, which indicated that the HMS might be composed of concentric multilayers (the observed wall thickness is ca. 40 nm). Furthermore, the marked red-shift of the ultraviolet–visible spectra suggested that J-aggregates had been formed (Fig. 3h)^46^, indicating that the β-CARs are oriented in the WCC bilayer with the long axis almost perpendicular to the bilayer surface and with the two WP5 located in the hydrophilic regions on both sides of the bilayer. Strong anisotropic photoluminescence (birefringence) was observed when the dispersion was placed between crossed polarizers (Fig. 3i, Supplementary Fig. 9), providing further evidence for the presence of ordered arrays of WP5 and β-CAR within the HMS. Transmission electron microscopy (TEM) was used to characterize the fine structure of the HMSs. Figure 3j shows a bright-field TEM micrograph of one HMS. The intensity of the central part of the HMS is much higher than that of the edge, indicating that the sphere is hollow. The thickness of the microspherical wall was calculated to be ∼100 nm based on this TEM image. The extended geometries of the β-CAR and their 1:1 complexation with the WCC are depicted Fig. 3k. Given that the maximal length of the WCC-based bilayer calculated using Gaussian is ∼4.6 nm, this TEM observation suggests that the HMS may possess a multilayer structure. Notably, WP5s with ten carboxylic acids possess negative charges, which might result in the enhancement of electrostatic repulsion. However, similar to the multiwalled microtubule assemblies obtained in weakly acidic environments^47^,^48^, the carboxylic acid groups in this study are partially dissociated in weakly basic environments. Hydrogen bonds thus dominate the interactions within the HMS and further weaken the electrostatic repulsion, facilitating the formation of multilayer microspheres. The packing information with respect to the WCC was obtained from small-angle X-ray scattering (SAXS) data. As shown in Fig. 3l, a broad Bragg reflection peak centred at 0.20 Å^−1^, corresponding to the ordered structure with an interlayer spacing of ∼3.1 nm, was observed. Note that this broad peak covers the *q* region range from 0.15 to 0.25 Å^−1^, corresponding to an interlayer spacing between 2.5 to 4.2 nm.

These distances were slightly shorter than the maximal calculated value (4.6 nm), possibly due to the conformational change or partial curling of the unsaturated carbon chain^49^. In addition, two slight peaks at lower *q* values (0.05 and 0.1 Å^−1^) were observed in the profile, indicating that there are multiple orders of structure with an interlayer spacing of ∼3.1 nm in the HMS. However, these peaks are weak, possibly because of the partially inhomogeneous lamellar structure within the HMS. In addition, considering the SAXS results, we conjectured that an interlayer spacing centred at 3.1 nm may exist in the HMS. Furthermore, the zeta-potential of the HMS decreased from ∼0 mV (pure β-CAR) to −31.6 mV, which provides further evidence that the hydrophilic WP5 is located on the exterior surface of the HMS (Supplementary Fig. 10).

On the basis of the aforementioned results, we proposed the following mechanism for the formation of the WCC-based HMSs (Fig. 3m): The hydrophobic interactions between β-CAR and WP5 provide the driving force for the formation of host–guest complexation WCC, which results in the phase transfer of β-CAR from ethanol to water. Meanwhile, hydrogen bonding (CH—O) and the CH—π interaction^50^ between β-CAR and WP5 render the WCC stable in water. Subsequently, hydrophobic interactions associated with aromatic stacking^51^,^52^ drive the WCC to assemble into an HMS, in which the hydrophobic β-CARs are shielded from the water, while the hydrophilic WP5s are in contact with the aqueous environment.

### Synthesis of HMS-based LHC containing Chl-b

Additional experiments were carried out to investigate the possibility of reproducing similar ‘tadpole-like' host–guest complexes by using Chl-b instead of β-CAR. When Chl-b solution was added to WP5, the proton peaks H_3_ of the methylene moieties on both rims were split into quartets from singlets, likely because of the inclusion of Chl-b in the cavity of WP5, which affected the swinging of the constituent units and led to the loss of the internal symmetry of the H_c_ protons. However, different from the remarkably chemical shift (β-CAR) before and after the complexation, the chemical shift of Chl-b changed slightly (Supplementary Figs 11–13). Furthermore, as shown in Supplementary Fig. 14a–d, according to the fluorescence intensity change of Chl-b (monitored at 450 nm), the *K*_a_ of WP5⊃Chl-b (WCB) was calculated to be (3.46±0.14) × 10^3^ M^−1^ (Supplementary Fig. 14e). The above results indicate that although Chl-b could thread into the cavity, the size mismatch between alkyl and cavity facilitates the alkyl in protruding from the cavity freely and thus could not result in the occupation of the cavity. The possible aggregates based on the unstable complexation was investigated, and no notable hierarchical nanostructure was observed when Chl-b was added to the solution of WP5 by using a similar method for HMS construction (Supplementary Fig. 15). Thus, based on the different binding affinities between WP5⊃β-CAR and WCB in binary solvents, Chl-b could play a role as a co-assembly component instead of a competitive guest during LHCs-b construction.

**Figure 4:**
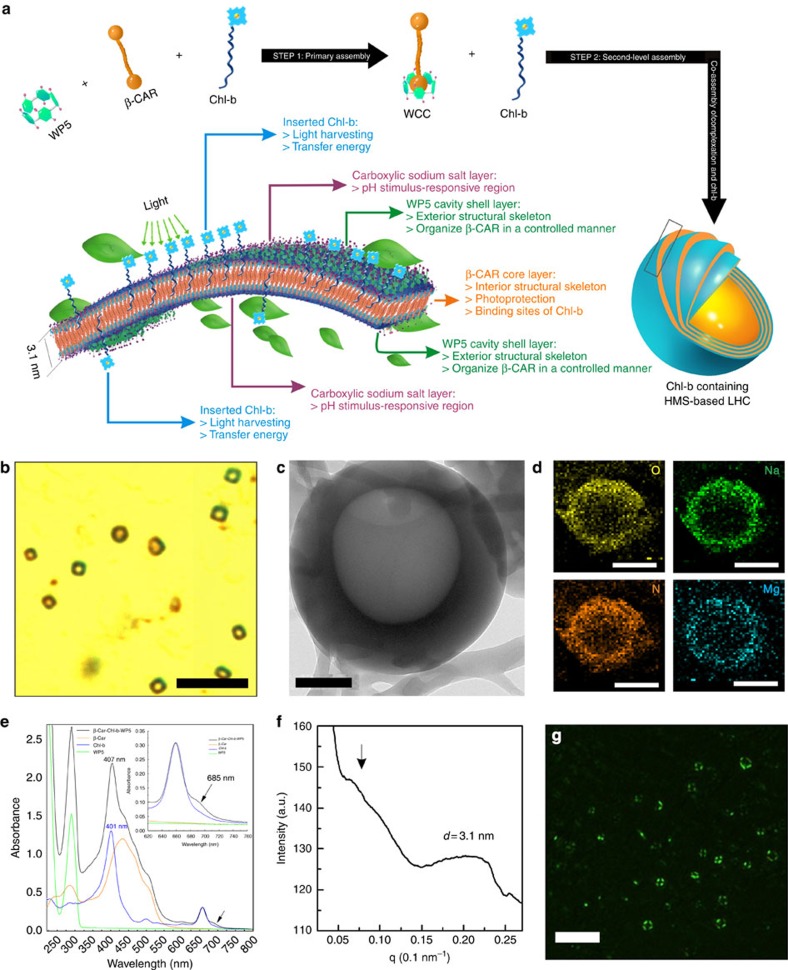
Synthesis of HMS-based LHCs-b. (**a**) Synthesis of LHCs-b. (**b**) OM image showing LHCs-b. Scale bar, 10 μm. (**c**) TEM image of an LHCs-b. Scale bar, 200 nm. (**d**) EDX mapping image of an LHCs-b (the colour in this image is false). Scale bar, 500 nm. (**e**) Ultraviolet–visible spectra of β-CAR, Chl-b, WP5 and LHCs-b. (**f**) SAXS profile of an LHCs-b. (**g**) Polarized image of LHCs-b (the colour in this image is false). Scale bar, 10 μm.

The route to synthesize the light-harvesting antenna is proposed in Fig. 4a. In the initial stage (step 1), the WCC is formed via host–guest recognition. According to the ^1^H-NMR results, WP5 is incapable of fixing Chl-b into a firm and durable cavity. Thus, once the cavity was filled by β-CAR, Chl-b had no opportunity to form complexation with WP5. In contrast, β-CAR could form complexation with WP5 when Chl-b protruded out of the cavity. Thus, WCC and Chl-b act as the main assembly factors. In this case, hydrophobic interactions further drive the WCC and Chl-b to assemble into LHCs containing Chl-b, in which the hydrophobic β-CAR and Chl-b tail are shielded from the water, whereas the hydrophilic WP5s and porphyrin are in contact with the aqueous environment (step 2). As in the LHCs containing Chl-b synthesis method, the β-CAR and Chl-b powders were first dissolved in ethanol, and then, 2 ml of the β-CAR and 2 ml of the Chl-b solutions were added to 4 ml of an aqueous solution of WP5 using a pipette, without stirring. After the solution was aged for 7 days, pale-orange aggregates appeared. Meanwhile, a clear Tyndall effect (Supplementary Fig. 16a) could be observed, indicating the formation of microaggregates. As shown in Fig. 4b, HMS-based LHCs containing Chl-b (LHCs-b) with orange–green-coloured walls were observed, confirming the homogeneous distribution of β-CAR and Chl-b throughout the LHCs-b. Compared with the narrow size distribution of HMSs (diameter range from 650 to 2,000 nm), the DLS results showed that the insertion of Chl-b causes the size distribution to become wider (Supplementary Fig. 16b). OM observation demonstrated that both LHCs-b with diameters <650 nm and >2,000 nm co-existed and also possessed hollow spherical structures (Supplementary Fig. 17). It is worth noting that some green liquid could be found in the cavities of LHCs-b through OM observation (blue arrows). It is speculated that a small amount of Chl-b did not participate in the wall construction and, as a result, was encapsulated in the cavities of the hollow spheres during the formation of the hollow spheres. Chl-b solution shows no photocatalytic activity and they were isolated by the cavities. Thus, no further experiment was designed to remove the non-aggregated Chl-b. The TEM image in Fig. 4c (Supplementary Figs 18,19) demonstrates that the hollow spherical structure was maintained after the insertion of Chl-b. The elemental mapping analysis demonstrates the homogeneous distribution of O and Na (the characteristic elements in the WP5), as well as the homogeneous distribution of N and Mg (the characteristic elements in Chl-b), across the entire LHC (Fig. 4d, Supplementary Fig. 19). The ultraviolet–visible spectra of LHCs-b showed a red-shifted shoulder (at 685 nm) absorption, which arose from alterations in the pigment–pigment interactions in LHCs-b (Fig. 4e).^53^ In addition, visible CD spectroscopy is a sensitive technique to monitor excitonic pigment–pigment and pigment–protein interactions^54^. As shown in Supplementary Fig. 20, the CD bands at (−)474, (−)488, (+)499, (−)653, (−)666, (+)702 and (+)724 nm were observed in LHCs-b. In the Q region, the CD spectra of the LHCs-b showed a negative peaks at (−)666 nm, which is characteristic of Chl-b^55^ and accompanied by negative peak at (−)653 nm. The positive peaks at (+)499 nm and (−)474 nm in the CD band were accompanied by a negative peak at (−)488 nm, originating from β-CAR^56^. Notably, the band at 649 nm (Chl-b in ethanol) was red-shifted to 666 nm with a shoulder at 653 nm on constitution into HMS. These results indicate that the Chl-b–Chl-b and/or Chl-b–β-CAR interactions were affected by constituting into HMS, which resulted from the changes in microenvironment of Chl-b in LHCs-b^57^. The bands at (−)474 and (+)499 nm as well as those at (−)666 nm and (+)724 nm may originate from excitonic interactions involving Chl-b and β-CAR molecules. The integrated areas of the positive and negative bands appear to be approximately equal^58^. These spectra suggest that in LHCs-b, a complicated set of excitonic interactions occurs between several Chl-b and β-CAR molecules^59^. Furthermore, the peak obtained from SAXS showed that the d-spacing remains at 3.1 nm (Fig. 4f), which provides further evidence that the ordered structure of the HMS was not destroyed after the co-assembly with Chl-b. In addition, the strong anisotropic photoluminescence shown in Fig. 4g is in good agreement with the SAXS data. Because the *K*_a_ of WP5⊃β-CAR was higher than that of WCB by two orders of magnitude, it could be deduced that almost all of the β-CAR in the system was wrapped by WP5. Thus, the position of β-CAR mainly located in the wall of the LHCs-b. Considering that hierarchical aggregates could not be obtained by WCB, a large amount of Chl-b, but not WCB randomly inserted into the HMS due to non-specific interactions. Therefore, it is hard to obtain a constant proportion of Chl-b among all LHCs-b (caused by the randomly insertion manner). As a result, it is difficult to obtain a convincing data by Elementary Analysis or Inductive Coupled Plasma Emission Spectrometer (proportion of Chl-b in each LHCs-b is different). Fortunately, EDX spectra provide a fine-testing method to investigate the β-CAR/Chl-b ratio in individual LHC-b. Thus, as shown in Supplementary Fig. 19a–d, LHCs-b with different diameter were selected and analysed. On the basis of the obtained semi-quantitative data, rough range of the Chl-b proportion could be provided (Supplementary Fig. 19a–d inset). Then, the average β-CAR/Chl-b ratio of ∼1.8 was obtained by calculation. Being confined to the accuracy of the semi-quantitative analysis (and interference of Chl-b solution in cavities), these data are provided only for providing a rough estimate.

### HMS-based LHCs served as photocatalytic entities

**Figure 5:**
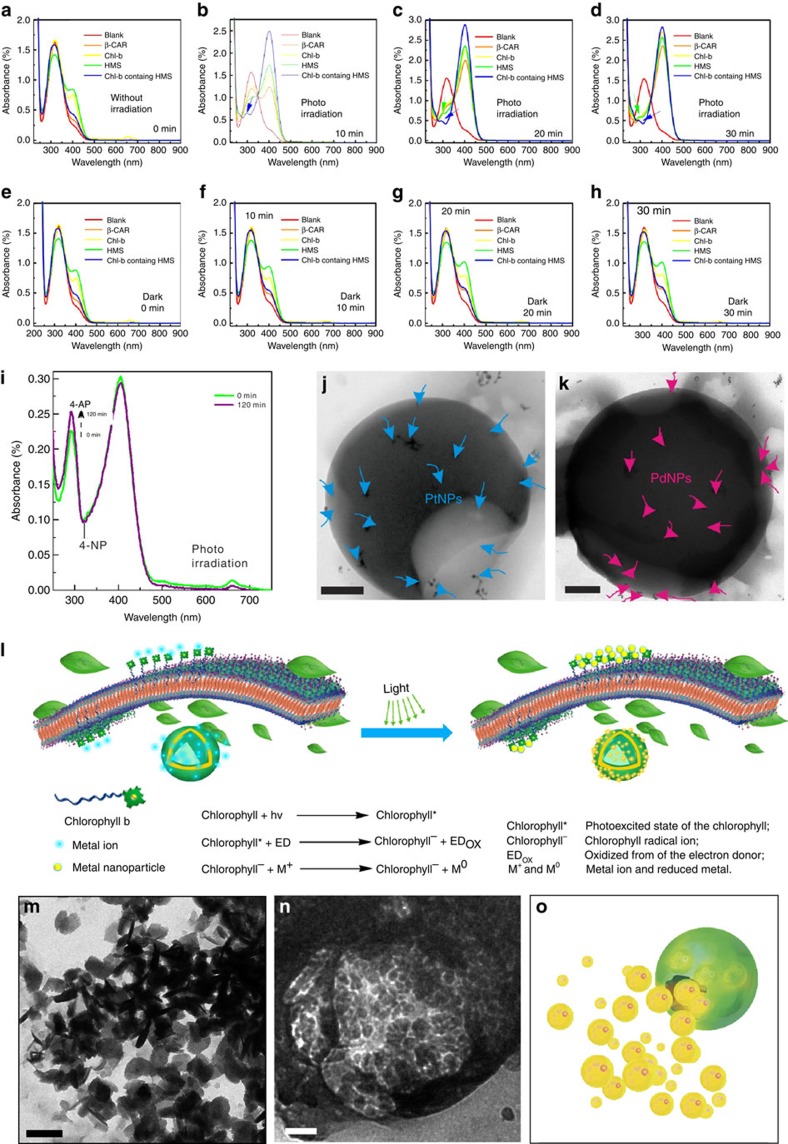
HMS-based LHCs-b served as photocatalytic entities. Ultraviolet–visible spectra of the transformation of 4-NP into 4-AP under photocatalysis by β-CAR, Chl-b, HMS, and LHCs-b for (**a**) 0 min, (**b**) 10 min, (**c**) 20 min and (**d**) 30 min. (**e**–**h**) are the corresponding untreated control groups for (**a**–**d**). (**i**) Ultraviolet–visible spectra of the transformation of 4-NP into 4-AP under photocatalysis by LHCs-b. TEM images of **j**, a PtNPs-loaded LHCs-b (the blue arrows indicate the PtNPs); Scale bar, 100 nm, and (**k**) a PdNPs-loaded LHCs-b (the pink arrows indicate the PdNPs); Scale bar, 200 nm. (**l**) Possible photosynthesis mechanism of LHCs-b. (**m**) TEM micrograph of a self-broken HMS during the HCl-triggered degradation. Scale bar, 500 nm. (**n**) TEM micrograph of an HMS containing AgNPs during the HCl-triggered degradation. Scale bar, 100 nm. (**o**) Schematic showing the release of nanoparticles.

The catalytic activity of LHCs-b was evaluated for the reduction of the pollutant 4-nitrophenol (4-NP) after the addition of NaNO_2_. Figure 5a shows the ultraviolet–visible spectra of 4-NP in ethanol/water solutions containing distilled water (blank sample), β-CAR, Chl-b, HMS and LHCs-b; in this case, the photoirradiation time was 0 min. All of these spectra display a main absorption at 318 nm (4-NP) and a shoulder peak at 400 nm (4-nitrophenolate ions). After irradiation with a mercury lamp (500 W) for 10 min (Fig. 5b), the peak intensity at 318 nm decreased and the peak intensity at 400 nm increased for all of the samples due to the formation of 4-nitrophenolate ions^60^. Importantly, a new peak at 290 nm was observed in the spectrum of the sample of LHCs-b (blue line), which indicated the generation of 4-aminophenol (4-AP) and further demonstrated the photocatalytic activity of LHCs-b. Remarkably, after being irradiated with the mercury lamp for 20 min, the HMS sample also exhibited photocatalytic activity towards 4-NP; as shown in Fig. 5c, a new peak at 290 nm appeared in the HMS spectrum (green line).

These findings reveal that β-CAR suprastructures also exhibited catalytic activity. Reasonably, as the irradiation time was prolonged (Fig. 5d), a peak was also observed at 290 nm in the spectra of HMS and LHCs-b. As revealed by the literature, the carotene molecules form a layer between 0–40-mm deep under the insect's cuticle, putting them in the perfect position to capture the Sun's light. Similar to the literature, the hierarchical way in which the carotene molecules are arranged in our studies provides the possibility of photocatalytic activity by the HMS^61^,^62^. Control experiments using untreated samples were performed for the same samples at different time intervals in the absence of photoirradiation. As shown in Fig. 5e–h, no changes were observed in the ultraviolet–visible spectra compared with those of the experimental group (Fig. 5a–d). Subsequently, LHCs-b were irradiated for up to 120 min (in 5 min intervals, from 0–120 min). As shown in Fig. 5i, when a mixture of 4-NP and LHCs-b was irradiated, the intensity of the peak at 290 nm increased due to the generation of 4-AP, which further confirmed that the LHCs-b can be used as a photocatalyst for the reduction of organic species.

Excitingly, when an aqueous solution of K_2_PtCl_4_, ascorbic acid (as an electron donor) and LHCs-b was irradiated for 2 h, LHC microspheres decorated with platinum nanoparticles were observed (Fig. 5j). Similarly, photoreduction of an Ag^I^ salt occurred within 2 h of irradiation and produced AgNP-loaded spheres, as revealed by TEM (Supplementary Fig. 21). Palladium nanoparticles were similarly obtained (Fig. 5k). The photocatalytic reaction is believed to occur through two steps. First, Chl-b captures light to produce photoexcited states that are rapidly reduced by an electron donor. Then, the resulting Chl-b radical anion can be used as a catalyst to reduce various metal salts to their metallic state through successive light-harvesting and photochemical cycles (Fig. 5l)^63^. Microspheres with stimuli responsiveness are extremely important for potential applications. A decrease in pH induces marked damage in HMSs (Fig. 5m), which appears to be attributable to a transition from a microspherical structure to a micellar structure. Therefore, the loading and release of guest molecules in response to a decrease in pH was investigated. Here, WP5-functionalized AgNPs used as a model guest were encapsulated in the microsphere interior during assembly. When the solution pH was adjusted to produce acidic conditions, the release of AgNPs from the cavity of an HMS was clearly observed (Fig. 5n). Figure 5o shows a schematic of the release of loaded nanoparticles, demonstrating the possible application of this system for controlled release.

## Discussion

In previous reports of β-CAR with host β-cyclodextrin or calixarenes, β-cyclodextrin was used to encapsulate the bulky group of β-CAR^64^,^65^ and calixarenes were used as nanocontainers. The natural *β-*cyclodextrins are concave macrocycles capable of expressing molecular recognition via the formation of noncovalent inclusions with suitably sized hydrophobic guests in their cavities. These complexes are currently used in many applications, including enhancing the water solubility of hydrophobic guests, controlling the release of volatile guests and protecting labile guests from degradation promoted by external agents. As for the calixarenes, β-CAR molecules were simply loaded in the hydrophilic vector by spinning disk processing. In this sense, a study of the association between β-CAR and other hosts is very important because it could provide better binding sites for carotenoids, allowing improved structural control via higher binding specificity (Supplementary Discussion). The formation of the WCC was supported by the Raman and ^1^H-NMR spectra, which demonstrated that the binding site was in the terpene section of β-CAR based on the hydrophobic interactions. The generation of an HMS must be associated with the structural characteristics of the WCC; when either WP5 or β-CAR was removed from the water or ethanol, no HMS formation was observed (Supplementary Fig. 22), convincingly demonstrating that the WCC formed by WP5 and β-CAR is undoubtedly the critical factor for the construction of HMSs. Interestingly, HMSs can provide a better chemical system in terms of stability without the loss of bioavailability (Supplementary Figs 23,24). The HMS was observed to display characteristics that differ from those of traditional synthetic molecule-based suprastructures (Supplementary Fig. 25). Furthermore, the peak in the SAXS profile is weak as a consequence of the low concentration of HMSs. As shown in Supplementary Fig. 26, the intensity of the peak increased with increasing acquisition time.

As shown in Supplementary Fig. 27, LHC containing Chl-a could also be obtained based on WCC-based HMS. LHCs-b exhibit photocatalytic activity, as was demonstrated through a series of experiments. Interestingly, the HMSs also exhibit photoactivity, which differs from that of LHCs-b in that it exhibits slower kinetics.

The successful creation of HMS provides sophisticated strategies to generate carotenoids-based hierarchical model, which will result in innovative approaches to generate controllable supramolecular structures composed of carotenoids. The introduction of various biomolecules, could lead to the development of this supramolecular platform into diverse artificial biological cells for mimicking and optimizing photosynthetic systems, which would further provide a photosynthetic model and important tools for the investigation of the origins of the bioenergy system in living organisms.

## Methods

### General considerations

All chemical reagents were purchased from Aladdin and used without further purification. ^1^H-NMR spectra were recorded on a Bruker Advance 600 MHz spectrometer using D_2_O as the solvent. β-CAR and Chl-b were purchased from EKEAR (Shanghai). Raman spectra were measured in solution using an In Renishaw Via Raman spectrometer (*λ*_exc_=532 nm). For Raman measurements, aqueous dispersions were dried onto glass slides; the focus was then centred on an individual HMS for detection. Fluorescence spectra were recorded on an F-4500 spectrometer; the spectra were corrected against a photomultiplier and against the lamp intensity. The slit width of both monochromators was 5.0 nm. ultraviolet–visible absorption spectra of the photocatalytic experiment (the experimental group and the untreated control group) were recorded on a UV-2550 spectrophotometer. Ultraviolet–visible absorption spectra for assembly characterization and photocatalysis experiments (0–120 min) were recorded on a UV-2501 spectrophotometer. FT-IR spectra were collected using potassium bromide pellets on a TENSOR 27 spectrometer. Circular dichroism spectra were measured in a Jasco-815 spectropolarimeter. To compare the shift of each peak more clearly, the intensity of the 649 nm peak in the LHCs containing Chl-b was adjusted to the same intensity as the base of Chl-b. Dynamic light scattering and zeta-potential measurements were carried out on a Malvern Nanosizer S instrument at room temperature. OM and polarized OM images were obtained using a Leica Microsystems DM LM/P instrument. Fluorescence OM images were acquired on an Olympus IX 73 W. D. 27 mm with an inverted configuration. For OM, polarized OM and fluorescence OM, aqueous dispersions of orange aggregates were dried onto glass slides for observation. The TEM images were obtained using a Philips TECNAI-12 instrument with an accelerating voltage of 120 kV. For TEM, aqueous dispersions of HMSs and Chl-b-containing LHC***s*** were dried onto carbon-coated copper support grids. A Hitachi S-4800 field-emission scanning electron microscope was used to investigate the HMS. For SEM, aqueous dispersions of HMSs were dried onto silicon wafers. HRTEM and elemental mapping images were obtained using a Tecnai G2 F30 S-TWIN instrument. SAXS experiments were performed at room temperature on a NanoSTAR, Bruker-AXS, 30 W. For SAXS, dispersions of WCC-based HMSs and Chl-b-containing LHCs were loaded into special glass X-ray capillary tubes with an internal diameter of 1.5 mm.

### Synthesis of pillar[5]arene WP5

Carboxyl-modified pillar[5]arene WP5 was prepared by a modified literature procedure^38^ and was identified by ^1^H-NMR spectroscopy in D_2_O. ^1^H-NMR (D_2_O, 600 MHz, p.p.m.): δ 6.7 (s, 10 H), 4.28 (d, 20 H), 3.90 (s, 10 H).

### Characterization of chemical and biomolecular materials

β-CAR (purity≥97%) and Chl-b (purity≥95%) were purchased from EKEAR (Shanghai). WP5 was prepared by a modified literature procedure and was identified by ^1^H-NMR spectroscopy in D_2_O.

### WCC studies

For ^1^H-NMR characterization, β-CAR was dissolved in ethanol-d^6^ to produce a 150 μM solution (curve a in Supplementary Figs 1, 2, solvent: ethanol-d^6^). WP5 was dissolved in D_2_O to produce a 300 μM aqueous solution, and then, an equal volume of ethanol-d^6^ was added to the WP5 solution to produce a 150 μM binary solvent solution (curve c in Supplementary Figs 1, 2, solvent: ethanol-d^6^: D_2_O=1:1). The D_2_O aqueous solution of WP5 added to the ethanol-d^6^ solution to produce a 150 μM WP5 and β-CAR complexation solution (curve b in Supplementary Figs 1, 2, solvent: ethanol-d^6^: D_2_O=1:1). Clear ^1^H-NMR spectra could not be achieved when β-CAR ethanol-d^6^ solution was added by an equal volume of D_2_O due to the poor solubility and low concentration of β-CAR in water in the absence of WP5. Thus, to obtain clear spectra, ethanol-d^6^ instead of binary solvents (ethanol-d^6^: D_2_O=1:1) were used for the β-CAR ^1^H-NMR characterization and for further comparison. In addition, the comparison between β-CAR (ethanol-d^6^) and WCC (ethanol-d^6^: D_2_O=1:1) is closer to the solvent environment changes in the assembly process conditions. For fluorescence characterization, various concentrations of β-CAR in ethanol solution were added to an aqueous solution of WP5 to produce β-CAR**/**WP5 solutions with different molar ratios of 0, 0.2, 0.3, 0.6, 0.8, 1.2, 1.6, 2.0 and 2.5. The emission intensities of the WP5 solutions at various molar ratios were monitored by fluorescence spectroscopy (*λ*_ex_=268 nm). Using a non-linear curve-fitting method, the *K*_a_ of WP5⊃β-CAR was estimated.The non-linear curve fitting was based on the equation Δ*F*=(*ΔF*_∞_/[H]_0_) (0.5[G]_0_+0.5([H]_0_+1/*K*_a_)−(0.5 ([G]_0_^2^+(2[G]_0_(1/*K*_a_ −[H]_0_))+(1/*K*_a_+[H]_0_) ^2^) ^0.5^)) (1) (refs 37, 66). On addition of β-CAR, the fluorescence intensity of WP5 (monitored at 653 nm) was gradually quenched, and the association constant of WP5⊃β-CAR was calculated. *ΔF* is the fluorescence intensity changes at 653 nm at [G]_0_; *ΔF_∞_* is the fluorescence intensity changes at 653 nm when WP5 is completely complexed, [G]_0_ is the initial concentration of β-CAR, [H]_0_ is the fixed initial concentration of WP5.

### WP5 and Chl-b interaction studies

For ^1^H-NMR characterization, Chl-b was dissolved in ethanol-d^6^ to produce a 150 μM solution (curve a in Supplementary Figs 11–13, solvent: ethanol-d^6^). WP5 was dissolved in D_2_O to produce a 300 μM aqueous solution, and then, an equal volume of ethanol-d^6^ was added to WP5 solution to produce a 150 μM binary solvent solution (curve c in Supplementary Figs 11–13, solvent: ethanol-d^6^: D_2_O=1:1). The D_2_O aqueous solution of WP5 added to the ethanol-d^6^ solution to produce a 150 μM WP5 and Chl-b complexation solution (curve b in Supplementary Figs 11–13, solvent: ethanol-d^6^: D_2_O=1:1). In accordance with the method used in previous experiments (WCC ^1^H-NMR characterization), ethanol-d^6^ was used for the Chl-b ^1^H-NMR characterization and for further comparison instead of binary solvents (ethanol-d^6^: D_2_O=1:1).

To determine the *K*_a_ for the complexation between WP5 and Chl-b, fluorescence titration experiments were carried out in solutions that had a constant concentration of Chl-b and varying concentrations of WP5. Various concentrations of WP5 solutions were added to an ethanol solution of Chl-b at the concentration of 100 μM (1,000 μl) to produce WP5**/**Chl-b solutions with different molar ratios (0, 0.2, 0.4, 0.6, 0.8, 1.0, 1.2, 1.4, 1.6, 2.2, 2.6 and 2.8). In a typical experiment, 1,000 μl of WP5 at the concentration of 20 μM were added to the Chl-b solution to produce WP5**/**Chl-b solutions with a molar fraction of 0.2. Then, 1,000 μl of WP5 at the concentration of 40 μM were added to the Chl-b solution to produce WP5**/**Chl-b solutions with a molar fraction of 0.4. Subsequently, 1,000 μl of WP5 at the concentration of 60 μM were added to the Chl-b solution to produce WP5**/**Chl-b solutions with a molar fraction of 0.6. The solutions with the other molar ratios (0.8, 1.0, 1.2, 1.4, 1.6, 2.2, 2.6 and 2.8) were prepared by a similar method. The emission intensities of the WP5 solutions at various molar ratios were monitored by fluorescence spectroscopy (*λ*_ex_=268 nm). The non-linear curve fitting was based on the equation Δ*F*=(*ΔF*_∞_/[G]_0_) (0.5[H]_0_+0.5([G]_0_+1/*K*a)−(0.5 ([H]_0_^2^+(2[H]_0_(1/*K*_a_ −[G]_0_))+(1/*K*_a_+[G]_0_) ^2^) ^0.5^)) (2) (refs 37, 66), which is was obtained by a modified literature equation (fix the concentration of guest and increase the concentration of host). According to the different emission peaks of WP5 and Chl-b in the range from 300 to 750 nm, the peak at 450 nm was selected to monitor the intensity change of Chl-b. On the basis of the fluorescence intensity change of Chl-b (monitored at 450 nm), the association constant of WCB was calculated. *ΔF* is the fluorescence intensity change at 450 nm at [H]_0_, *ΔF_∞_* is the fluorescence intensity change at 450 nm when WP5 is completely complexed; [G]_0_ is the fixed initial concentration of Chl-b; [H]_0_ is the initial concentration of WP5.

### Synthesis of HMSs

WP5 was first basified with sodium hydroxide to produce an aqueous solution. Then, β-CAR was dissolved in ethanol to produce an ethanol solution of β-CAR. Subsequently, β-CAR-based HMSs were prepared by pipetting an ethanol solution of β-CAR into an aqueous solution of WP5 and then aging. The final concentration of host and guest could be controlled by adjusting their initial concentration. In a typical experiment, HMSs were prepared by pipetting 2 ml of an ethanol solution of β-CAR (150 μM) into 2 ml of an aqueous solution of WP5 (150 μM) and then aging the solution for 7 days at room temperature.

### Synthesis of LHCs-b

WP5 was first basified with sodium hydroxide to produce an aqueous solution. Then, β-CAR was dissolved in ethanol to produce an ethanol solution of β-CAR. Chl-b was dissolved in ethanol to produce an ethanol solution of Chl-b. Simlar to the synthesis method of HMSs, LHCs-b were prepared at room temperature by pipetting an ethanol solution of β-CAR and Chl-b into an aqueous solution of WP5. The final concentration of host and guest could be controlled by adjusting their initial concentration. In a typical experiment, LHCs-b were prepared at room temperature by pipetting 2 ml of an ethanol solution of β-CAR (150 μM) and 2 ml of an ethanol solution of Chl-b (150 μM) into 4 ml of an aqueous solution of WP5. Mixing the solution resulted in a pale-orange turbid suspension that was subsequently aged for 7 days.

### Photocatalytic synthesis of metal nanoparticles

In a typical experiment, 0.50 ml of a suspension of LHCs-b, 20 μl of aqueous K_2_PtCl_4_ (10 mM) and 25 μl of aqueous ascorbic acid (0.2 M) were sequentially added to a 2-ml glass vial and then irradiated for 2 h with visible light from a 300-W Xe lamp. Pd and Ag nanoparticles were prepared using similar methods.

### Photocatalytic reduction of 4-NP

*Experimental group*. Blank sample: 1 ml of 4-NP stock solution (2 mM), 100 μl of sodium nitrite stock solution (0.1 M) and 300 μl of distilled water were mixed and homogenized in a glass vial and then irradiated with visible light from a 500-W mercury lamp. β-CAR sample: One millilitre of 4-NP stock solution (2 mM), 100 μl of sodium nitrite stock solution (0.1 M) and 300 μl of a dispersion of β-CAR (75 μM) were mixed and homogenized in a glass vial and then irradiated with visible light from a 500-W mercury lamp. Chl-b sample: 1 ml of 4-NP stock solution (2 mM), 100 μl of sodium nitrite stock solution (0.1 M) and 300 μl of a dispersion of Chl-b (75 μM) were mixed and homogenized in a glass vial and then irradiated with visible light from a 500-W mercury lamp. HMS sample: 1 ml of 4-NP stock solution (2 mM), 100 μl of sodium nitrite stock solution (0.1 M) and 300 μl of a dispersion of HMS (75 μM) were mixed and homogenized in a glass vial and then irradiated with visible light from a 500-W mercury lamp. LHCs-b sample: 1 ml of 4-NP stock solution (2 mM), 100 μl of sodium nitrite stock solution (0.1 M) and 300 μl of a dispersion of LHCs-b (75 μM) were mixed and homogenized in a glass vial and then irradiated with visible light from a 500-W mercury lamp.

*Untreated control group*. Blank sample: 1 ml of 4-NP stock solution (2 mM), 100 μl sodium nitrite stock solution (0.1 M) and 300 μl of distilled water were mixed and homogenized in a glass vial and then stored in the dark. β-CAR sample: 1 ml of 4-NP stock solution (2 mM), 100 μl sodium nitrite stock solution (0.1 M) and 300 μl of a dispersion of β-CAR (75 μM) were mixed and homogenized in a glass vial and then stored in the dark. Chl-b sample: 1 ml of 4-NP stock solution (2 mM), 100 μl sodium nitrite stock solution (0.1 M) and 300 μl of a dispersion of Chl-b (75 μM) were mixed and homogenized in a glass vial and then stored in the dark. HMS sample: 1 ml of 4-NP stock solution (2 mM), 100 μl sodium nitrite stock solution (0.1 M) and 300 μl of a dispersion of HMS (75 μM) were mixed and homogenized in a glass vial and then stored in the dark. LHCs-b: 1 ml of 4-NP stock solution (2 mM), 100 μl sodium nitrite stock solution (0.1 M) and 300 μl of a dispersion of LHCs-b (75 μM) were mixed and homogenized in a glass vial and then stored in the dark. Changes in the absorption peaks over time associated with the photocatalytic reduction of 4-NP to 4-AP (experimental group and untreated control group) were monitored by ultraviolet–visible spectroscopy (UV-2501).

For the photocatalytic activity investigation of the LHCs-b sample using a longer duration of irradiation, the procedure was as follows: A 4-NP stock solution (2 mM), a sodium nitrite stock solution (0.1 M) and a dispersion of LHCs-b were mixed and homogenized in a glass vial and then irradiated with visible light from a 300-W Xe lamp. Changes in the absorption peaks over time associated with the photocatalytic reduction of 4-NP to 4-AP (0–120 min) were monitored by ultraviolet–visible spectroscopy (UV-2501).

### Data availability

The authors declare that the data supporting the findings of this study are available within the article and its Supplementary Information files, and all relevant data are available from the authors.

## Additional information

**How to cite this article:** Sun, Y. *et al.* Stimulus-responsive light-harvesting complexes based on the pillararene-induced co-assembly of β-carotene and chlorophyll. *Nat. Commun.* 7:12042 doi: 10.1038/ncomms12042 (2016).

## Supplementary Material

Supplementary Information Supplementary Figures 1-27, Supplementary Discussion and Supplementary References
