# Supplementary material for: Stimulus-responsive light-harvesting complexes based on the pillararene-induced co-assembly of β-carotene and chlorophyll
Source: Nat Commun. 2016 Jun 27;7:12042. doi: 10.1038/ncomms12042 (PMC4931247; doi:10.1038/ncomms12042)
Supplement: Supplementary Information — Supplementary Figures 1-27, Supplementary Discussion and Supplementary References [file ncomms12042-s1.pdf]

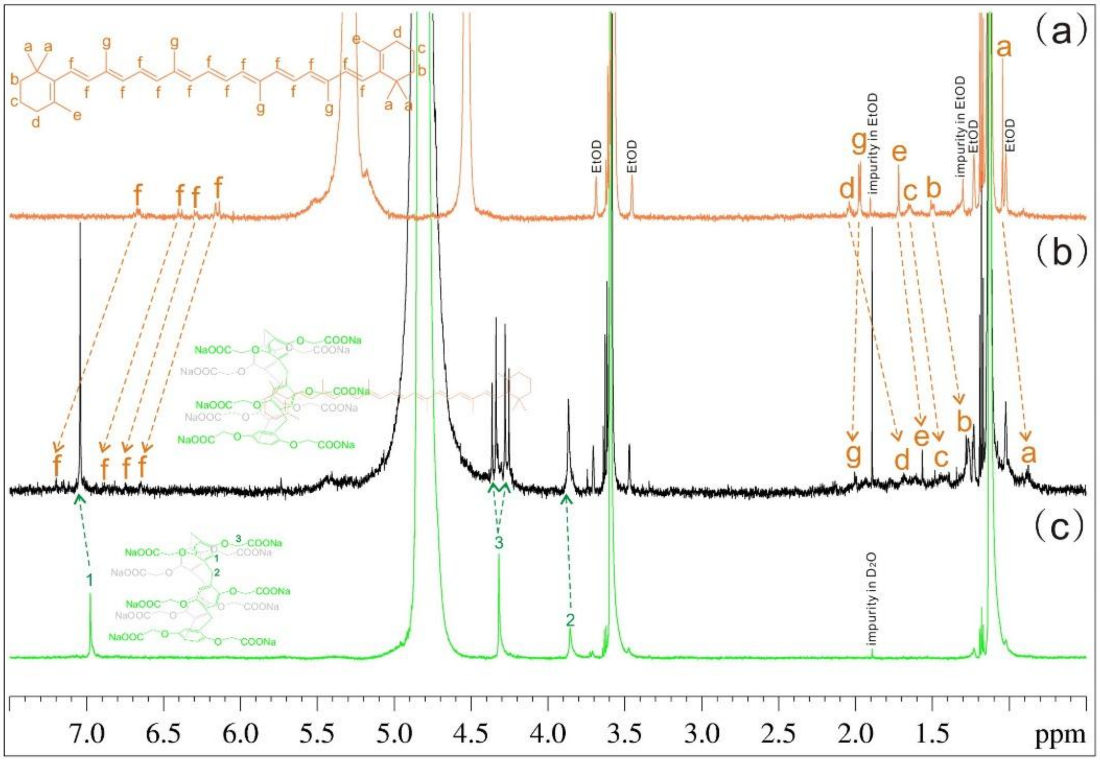

Supplementary Figure 1: ^{1}H-NMR of host and guest. (a) ^{1}H-NMR (600 Hz, Ethanol-d^6) spectra of ***β-CARs***[1] at a concentration of 150 μM, (b) ^{1}H NMR (600 Hz, D_2O/ Ethanol-d^6=1:1) spectra of ***β-CAR*** and ***WP5*** at a concentration of 150 μM, (c) ^{1}H NMR (600 Hz, D_2O/ Ethanol-d^6=1:1) spectra of ***WP5*** at a concentration of 150 μM.

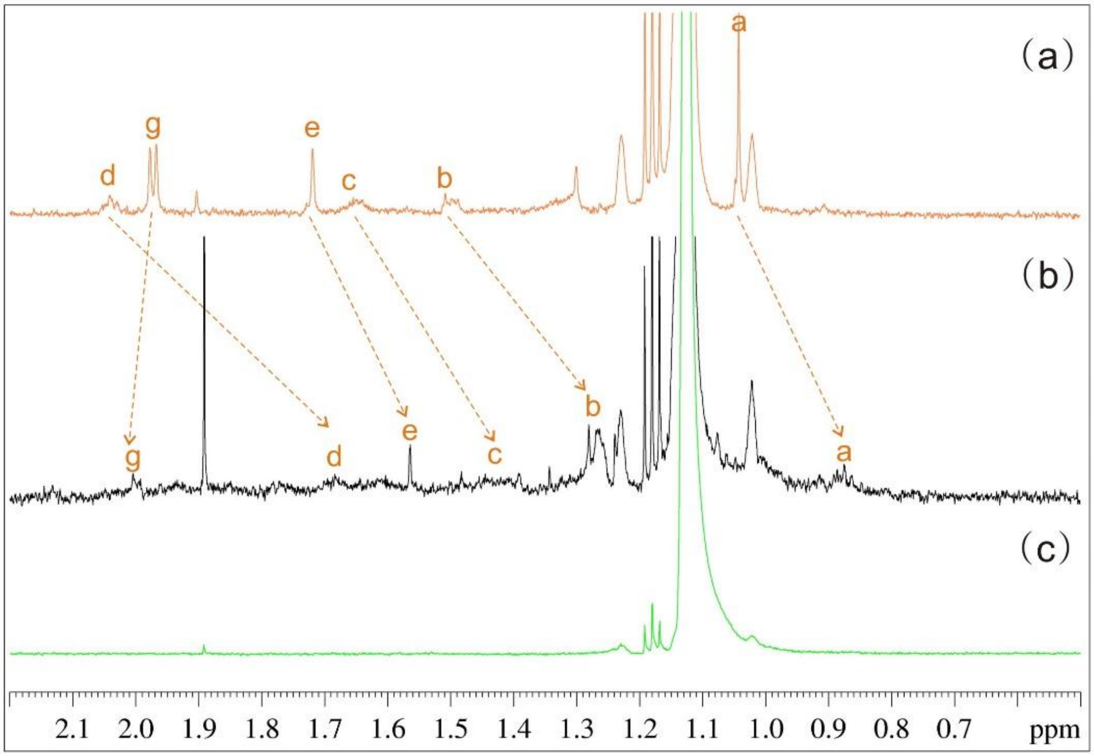

Supplementary Figure 2: ^{1}H-NMR of host and guest. (a) Partial ^{1}H-NMR (600 Hz, Ethanol-d^{6}) spectra of ***β-CAR*** at a concentration of 150 μM, (b) partial ^{1}H NMR (600 Hz, D_2O/ Ethanol-d^{6}=1:1) spectra of ***β-CAR*** and ***WP5*** at a concentration of 150 μM, (c) partial ^{1}H NMR (600 Hz, D_2O/ Ethanol-d^{6}=1:1) spectra of ***WP5*** at a concentration of 150 μM.

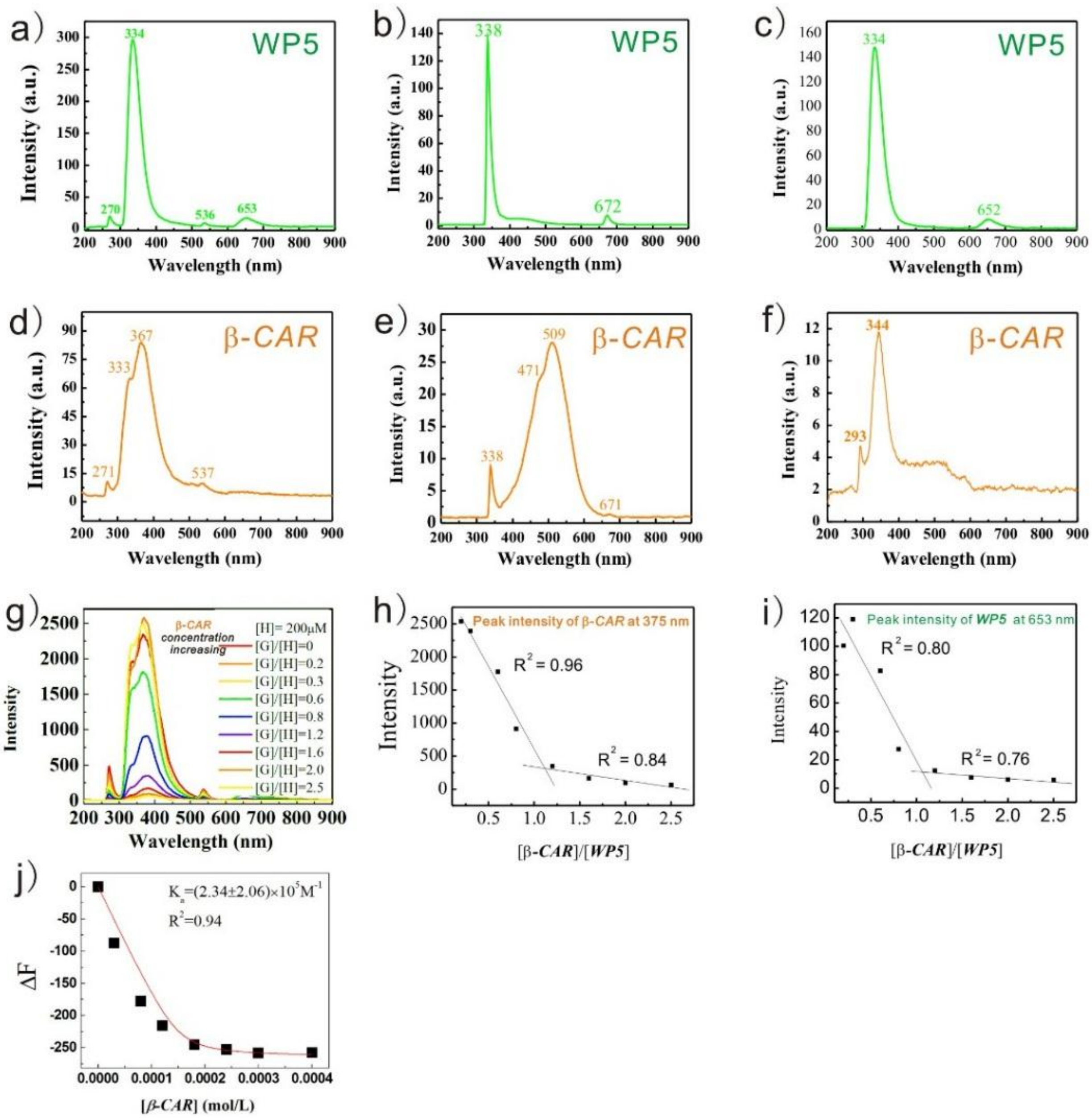

Supplementary Figure 3: Fluorescence of host, guest and complex. Fluorescence spectra of ***WP5*** (200 μM) in aqueous solution **(a)** excited at 268 nm, **(b)** excited at 290 nm, and **(c)** excited at 336 nm. Fluorescence spectra of ***β-CAR*** (200 μM) in ethanol **(d)** excited at 268 nm, **(e)** excited at 290 nm, and **(f)** excited at 336 nm. **(g)** Fluorescence spectra of ***WP5*** (200 μM) upon addition of ***β-CAR*** in ethanol/water (excited at 268 nm) at room temperature. **(h)** The fluorescence intensity changes of ***β-CAR***. **(i)** The fluorescence intensity changes of ***WP5***. **(j)** The fluorescence intensity changes of ***WP5*** upon addition of ***β-CAR***. The red solid line was obtained from the non-linear curve-fitting using eq. 1.

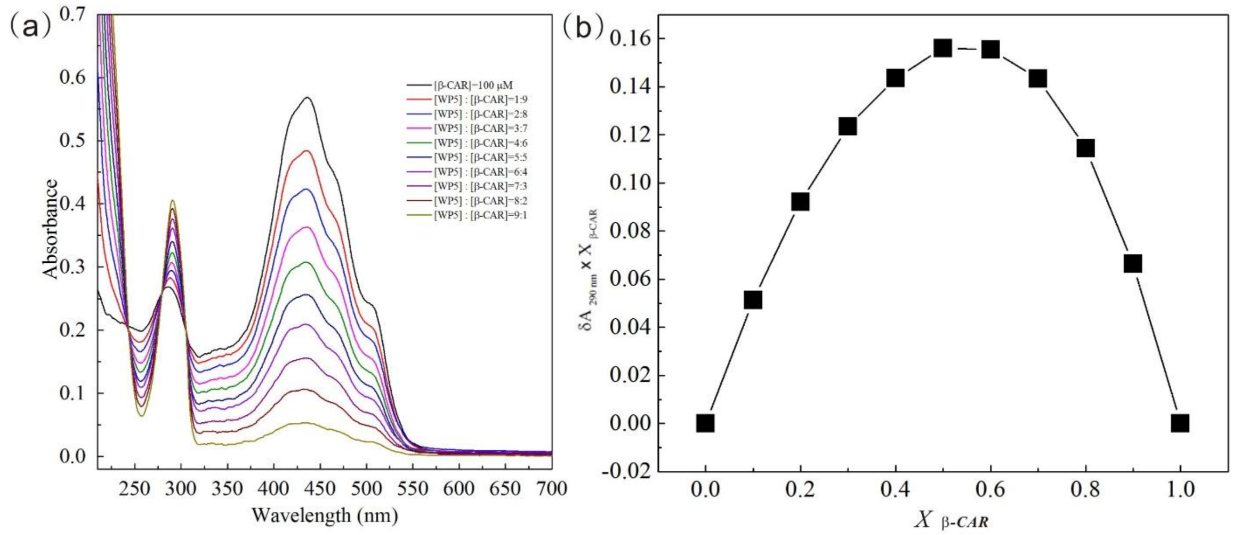

Supplementary Figure 4: UV-Vis absorption spectra and job plot of complex *WP5⊃β-CAR*. (a) UV-Vis absorption spectra of complex ***WP5⊃β-CAR*** with different molar ratios in water/ethanol (1:1) with a constant concentration of [***WP5***] + [***β-CAR***] = 100 μM. (b) Job plot showing the 1:1 stoichiometry of the complex between ***WP5*** and ***β-CAR*** by plotting the difference in absorption at 436 nm (a characteristic absorption peak of ***β-CAR***) against the mole fraction of ***β-CAR*** at an invariant total concentration of ***β-CAR*** in aqueous solution.

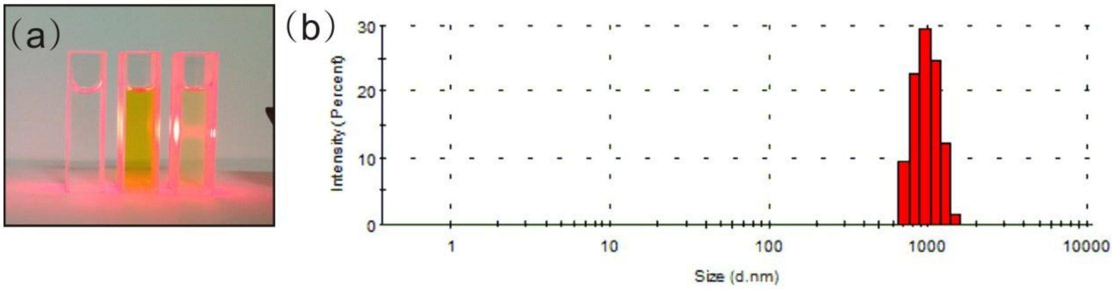

Supplementary Figure 5: Tyndall effect and DLS results. (a) Image of the Tyndall effect (left: the solution of ***WP5***, middle: the solution of ***β-CAR***, right: the solution of ***WP5*** and ***β-CAR*** aggregates). (b) DLS results of the ***WP5⊃β-CAR***-based ***HMS***.

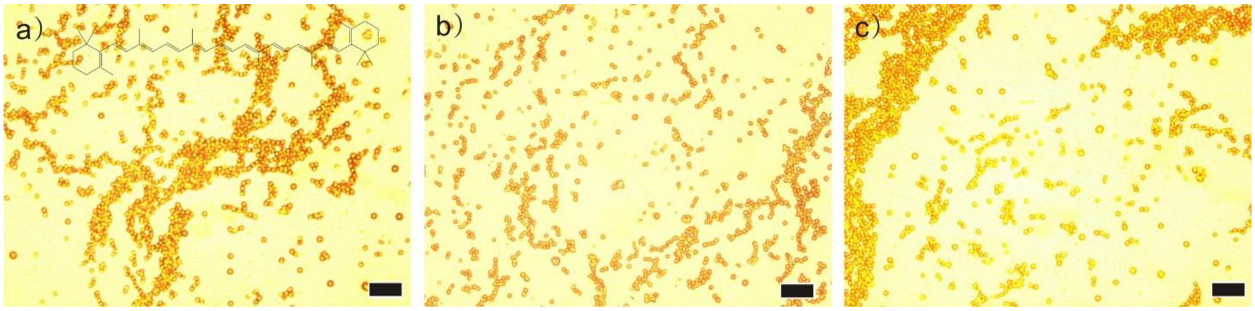

Supplementary Figure 6: Optical microscopy images. Optical microscopy images showing the ***HMS*** dispersion. Scale bar 10 μm.

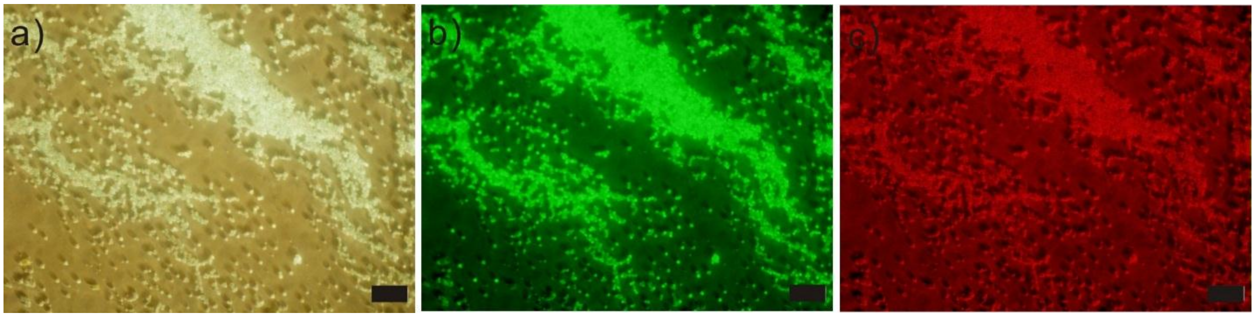

Supplementary Figure 7: Optical microscopy images. Fluorescence microscopy images showing autofluorescence after excitation with **(a)** UV light, **(b)** blue light, and **(c)** green light. Scale bar 10 μm.

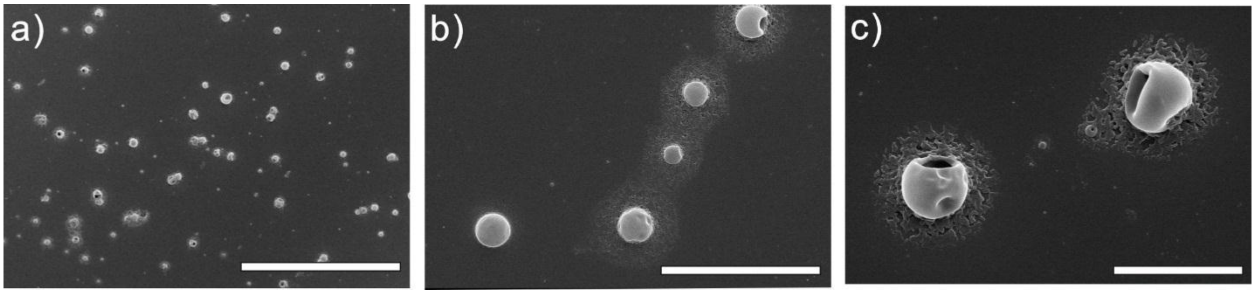

Supplementary Figure 8: SEM images. SEM images of ***HMS***s, including **(a)** the intensity observed over a large sample area, scale bar 50 μm, **(b)** an enlarged image of ***HMS***s, scale bar, 10 μm, and **(c)** an enlarged image of individual ***HMS***s. Scale bar 5 μm.

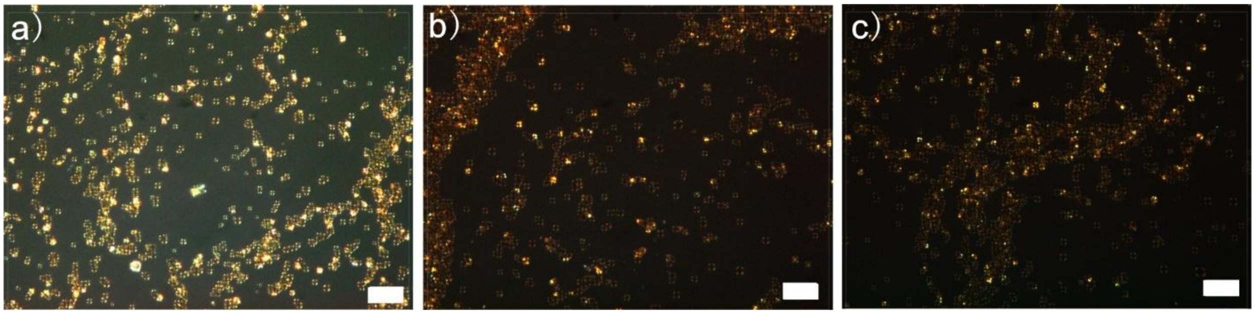

Supplementary Figure 9: Optical microscopy images. (a)-(c) Polarized images of ***HMS***s showing strong anisotropic photoluminescence attributed to birefringence when placed between crossed polarizers. Scale bar 10 μm.

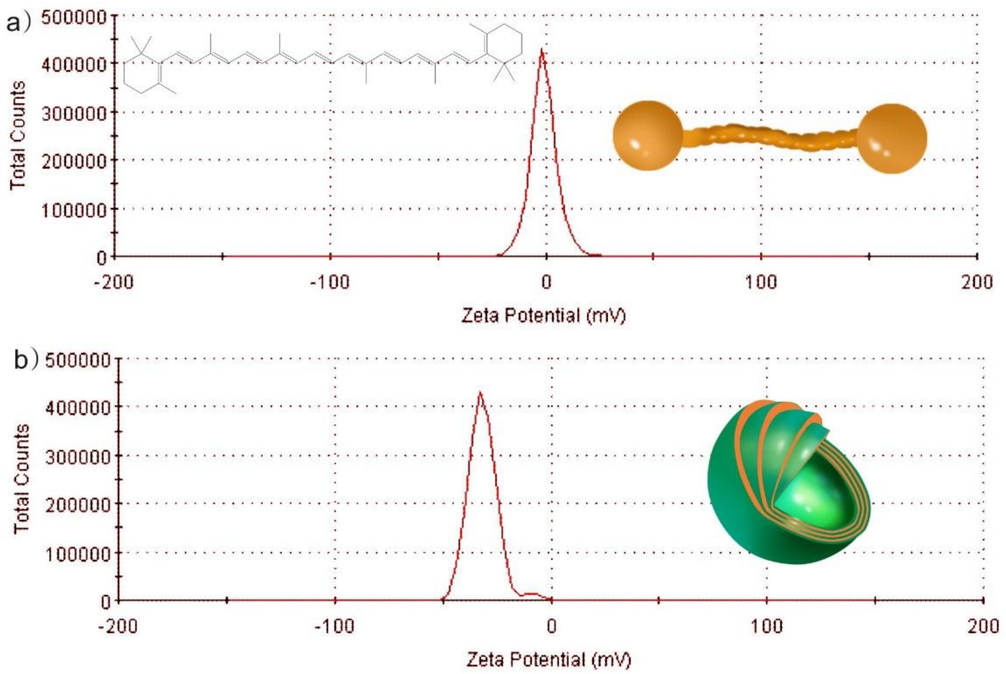

Supplementary Figure 10: Zeta potential experiments. Zeta potential of (a) ***β-CAR*** and (b) ***HMSs***.

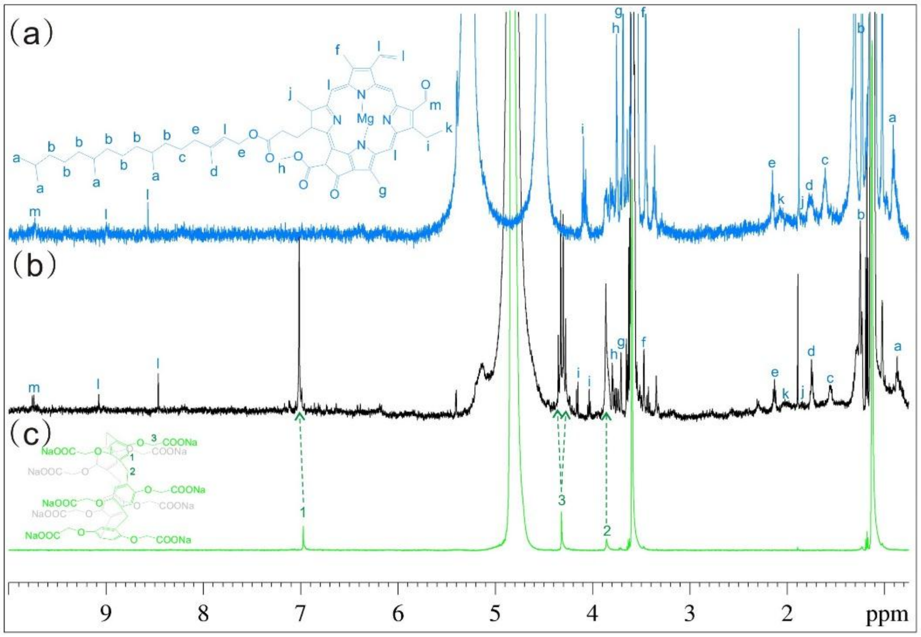

Supplementary Figure 11: ^{1}H-NMR of host and *Chl-b*. (a) ^{1}H-NMR (600 Hz, Ethanol-d^6) spectra of ***Chl-b*[2]** at a concentration of 150 μM, (b) ^{1}H NMR (600 Hz, D_2O/ Ethanol-d^6=1:1) spectra of ***Chl-b*** and ***WP5*** at a concentration of 150 μM, (c) ^{1}H NMR (600 Hz, D_2O/ Ethanol-d^6=1:1) spectra of ***WP5*** at a concentration of 150 μM.

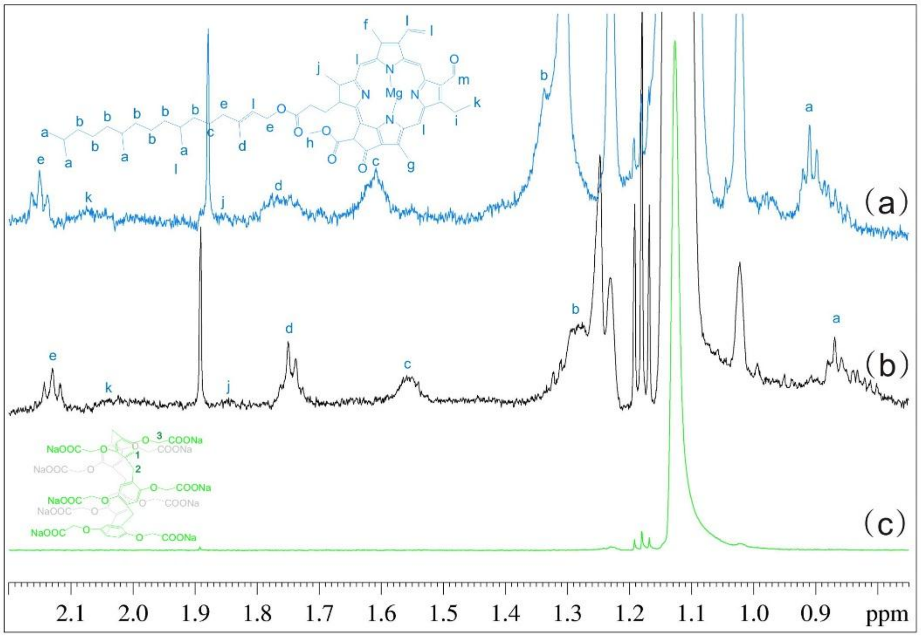

Supplementary Figure 12: ^{1}H-NMR of host and *Chl-b*. (a) Partial ^{1}H-NMR (600 Hz, Ethanol-d^6) spectra of ***Chl-b*** at a concentration of 150 μM, (b) partial ^{1}H NMR (600 Hz, D_2O/ Ethanol-d^6=1:1) spectra of ***Chl-b*** and ***WP5*** at a concentration of 150 μM, (c) partial ^{1}H NMR (600 Hz, D_2O/ Ethanol-d^6=1:1) spectra of ***WP5*** at a concentration of 150 μM.

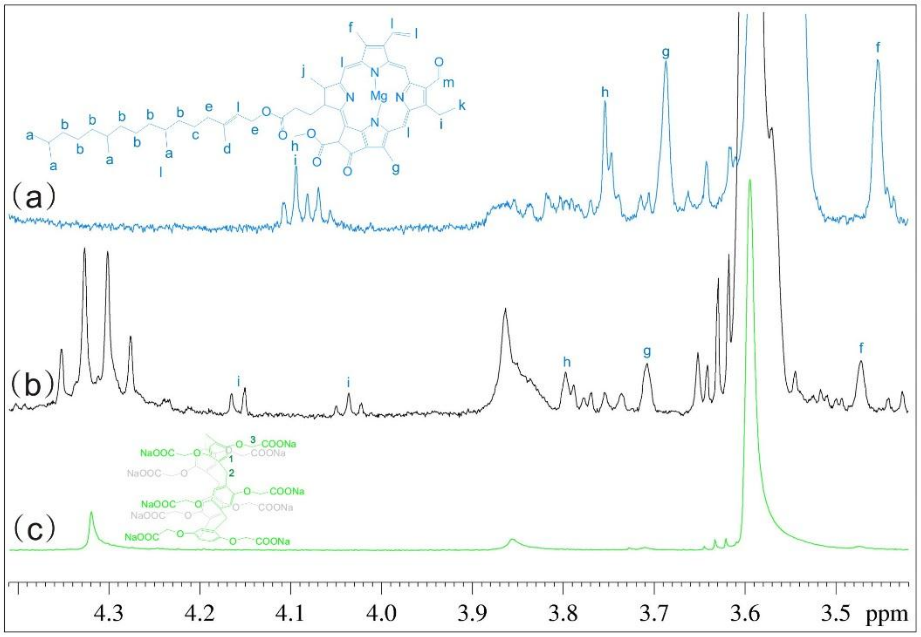

Supplementary Figure 13: ^{1}H-NMR of host and *Chl-b*. (a) Partial ^{1}H-NMR (600 Hz, Ethanol-d^6) spectra of ***Chl-b*** at a concentration of 150 μM, (b) partial ^{1}H NMR (600 Hz, D_2O/ Ethanol-d^6=1:1) spectra of ***Chl-b*** and ***WP5*** at a concentration of 150 μM, (c) partial ^{1}H NMR (600 Hz, D_2O/ Ethanol-d^6=1:1) spectra of ***WP5*** at a concentration of 150 μM.

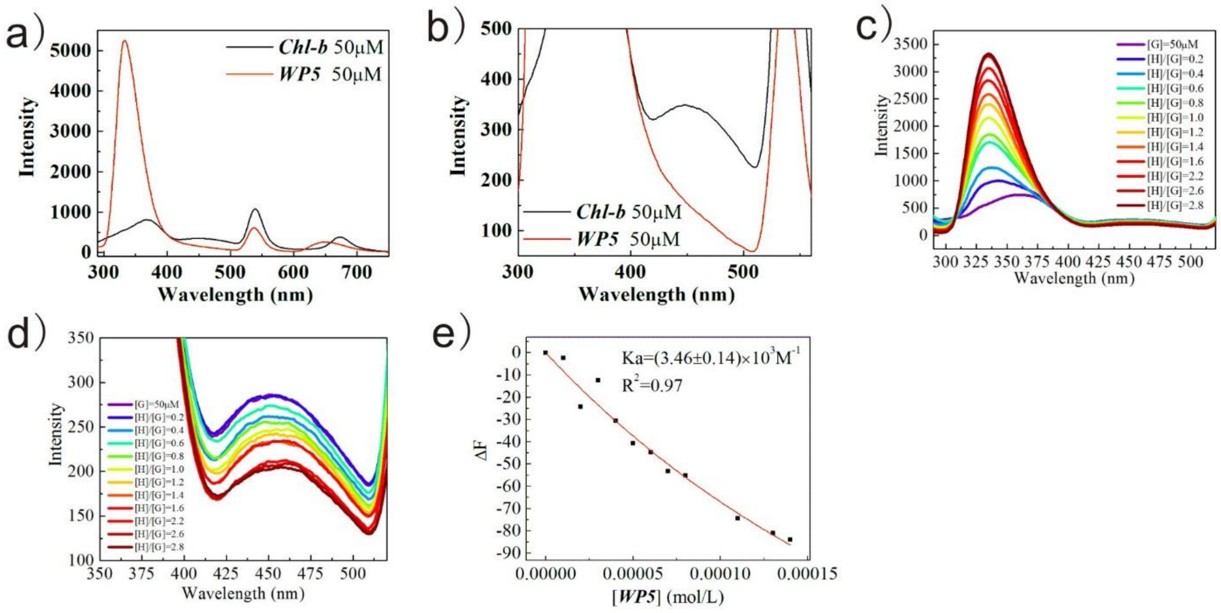

Supplementary Figure 14: Fluorescence spectra of *WP5*, *Chl-b* and the complexes. Fluorescence spectra of **(a)** ***WP5*** (50 μM) in aqueous solution and ***Chl-b*** (50 μM) in ethanol (excited at 268 nm), **(b)** magnified fluorescence of a, (**c**) fluorescence spectra of ***WP5*** (0-140 μM) in binary solvents (excited at 268 nm). Upon addition of ***WP5***, emission from ***Chl-b*** was quenched, indicating the formation of the ***WP5-Chl-b*** complex. (**d**) The fluorescence intensity changes of ***Chl-b*** upon addition of ***WP5***. The solid line was obtained from the non-linear curve-fitting using eq. 2.

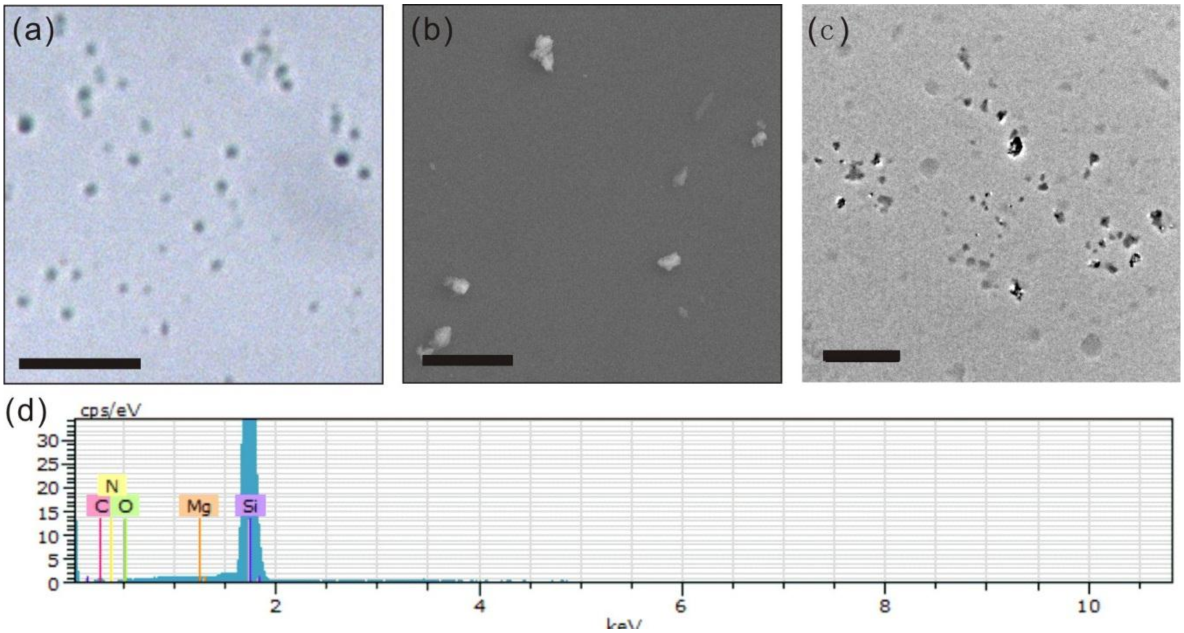

Supplementary Figure 15: *Chl-b* and *WP5* aggregates. (a) Optical microscopy image of ***Chl-b*** and ***WP5*** aggregates in the absence of ***β-CAR*** (final concentrations: [***WP5***]=75 μM, [***Chl-b***]=75 μM). Scale bar 10 μm. (b) SEM image of ***Chl-b*** and ***WP5*** aggregates in absence of ***β-CAR*** (final concentrations: [***WP5***]=75 μM, [***Chl-b***]=75 μM). Scale bar 10 μm. (c) TEM image of ***Chl-b*** and ***WP5*** aggregates in the absence of ***β-CAR*** (final concentrations: [***WP5***]=75 μM, [***Chl-b***]=75 μM). Scale bar 1 μm. (d) Energy spectrum of ***Chl-b*** and ***WP5*** aggregates in the absence of ***β-CAR*** (final concentrations: [***WP5***]=75 μM, [***Chl-b***]=75 μM).

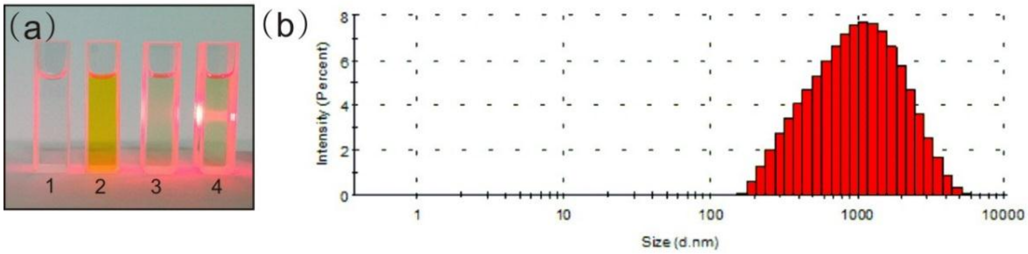

Supplementary Figure 16: Tyndall effect and DLS results. (a) Image of the Tyndall effect (1: the solution of ***WP5*** at a concentration of 75 μM, 2: the solution of ***β-CAR*** at a concentration of 75 μM, 3: the solution of ***Chl-b*** at a concentration of 75 μM, 4: the solution of ***LHCs*** containing ***Chl-b***). (b) DLS results of ***LHCs*** containing ***Chl-b***.

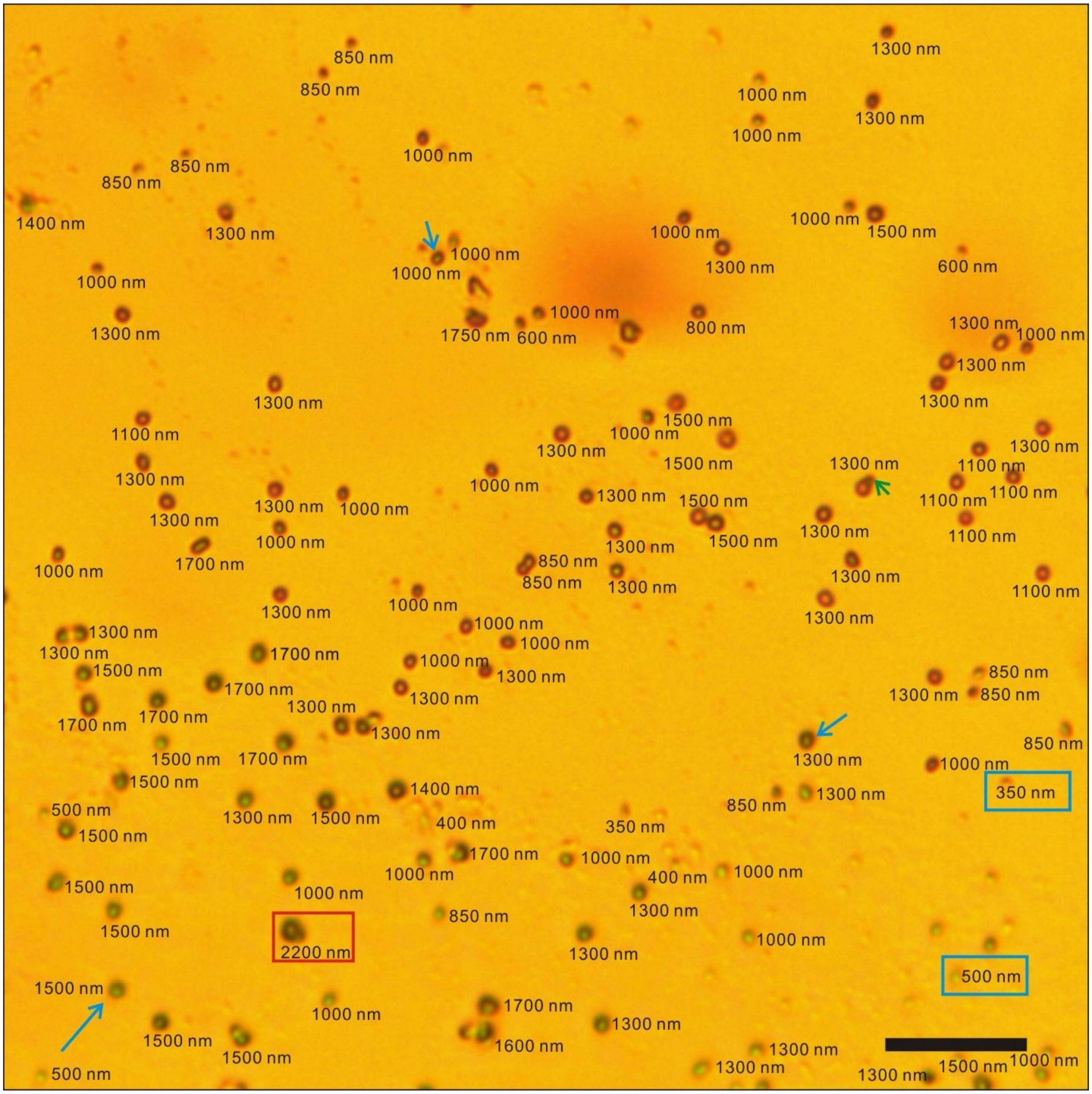

Supplementary Figure 17: Optical microscopy image of *HMS*-based *LHCs-b*. Scale bar 10 μm.

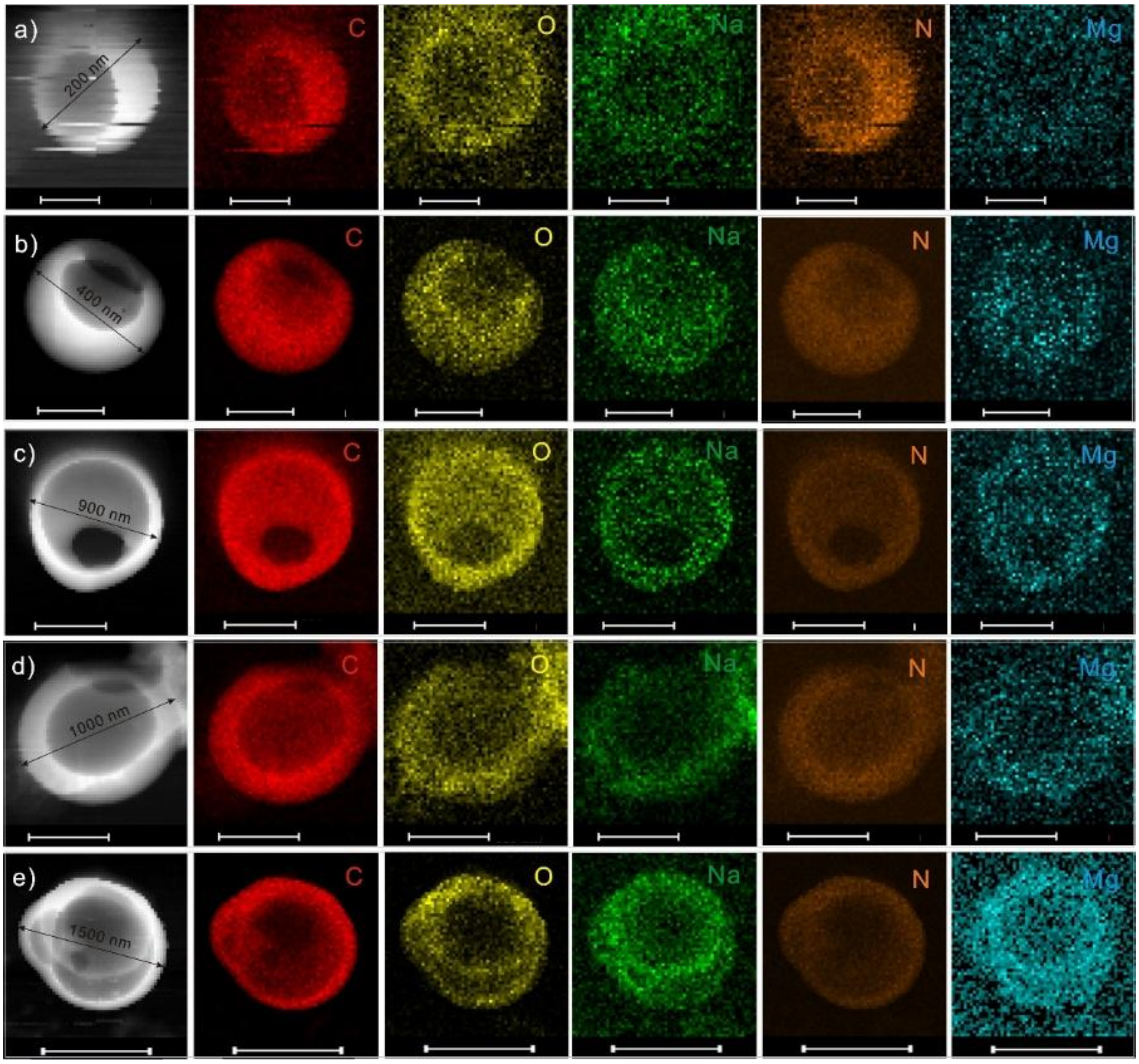

Supplementary Figure 18: EDX mapping image of an ***LHC*** containing ***Chl-b***. *LHC* containing *Chl-b* with **(a)** diameter of 200 nm. Scale bar 100 nm, **(b)** diameter of 400 nm. Scale bar 200 nm, **(c)** diameter of 900 nm. Scale bar 500 nm, **(d)** diameter of 1000 nm. Scale bar 500 nm, **(e)** diameter of 1500 nm. Scale bar 1 μm.

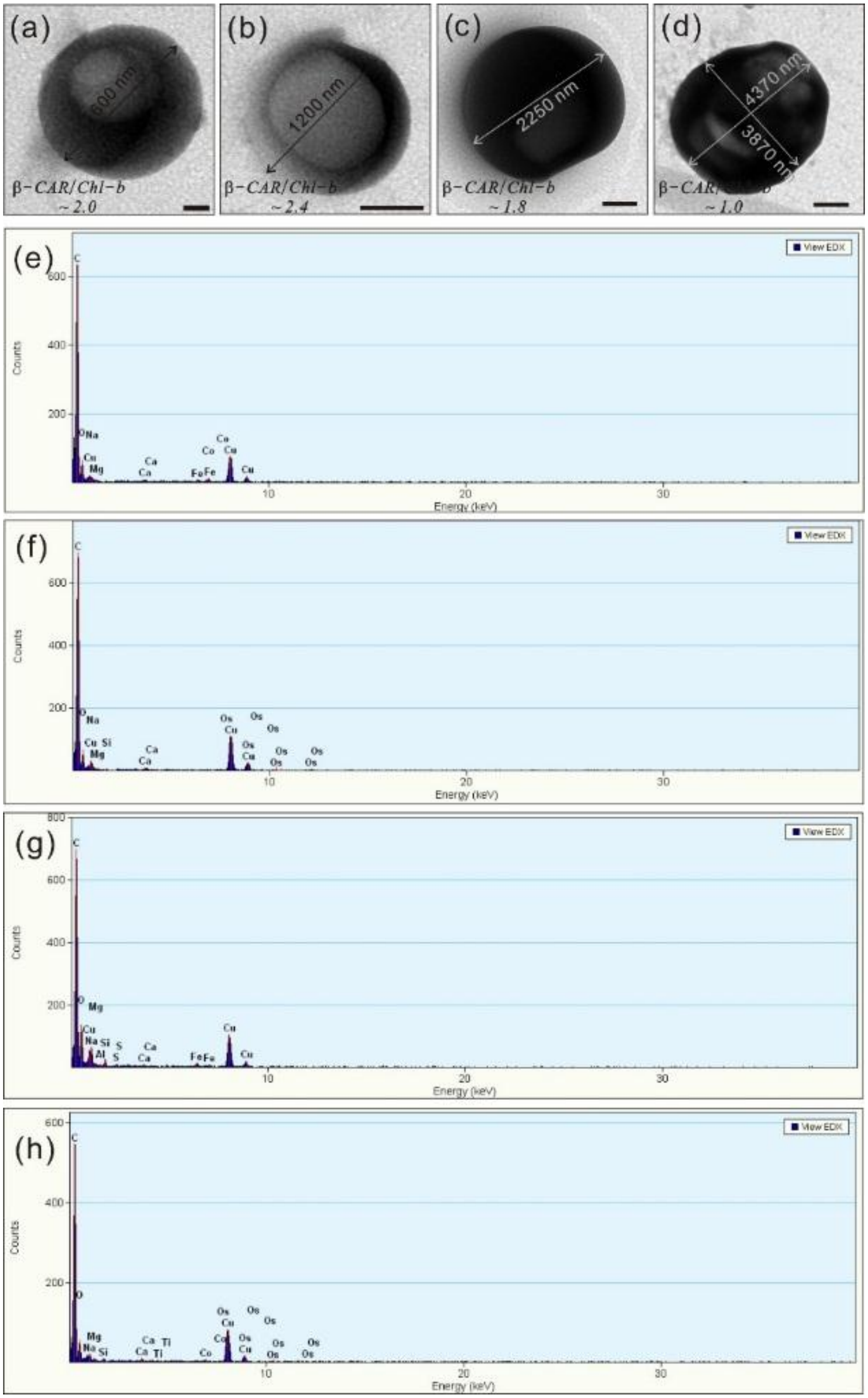

Supplementary Figure 19: TEM image of *HMS*-based *LHCs-b* with diameter of (a) 600 nm**.** Scale bar, 100 nm. (b) 1200 nm**.** Scale bar, 500 nm. (c) 2250 nm**.** Scale bar, 500 nm. (d) 4370 nm**.** Scale bar, 1000 nm. (e-f) Corresponding EDX spectra of ***LHC**s-**b***. These semi-quantitative analysis data are provided for reference only.

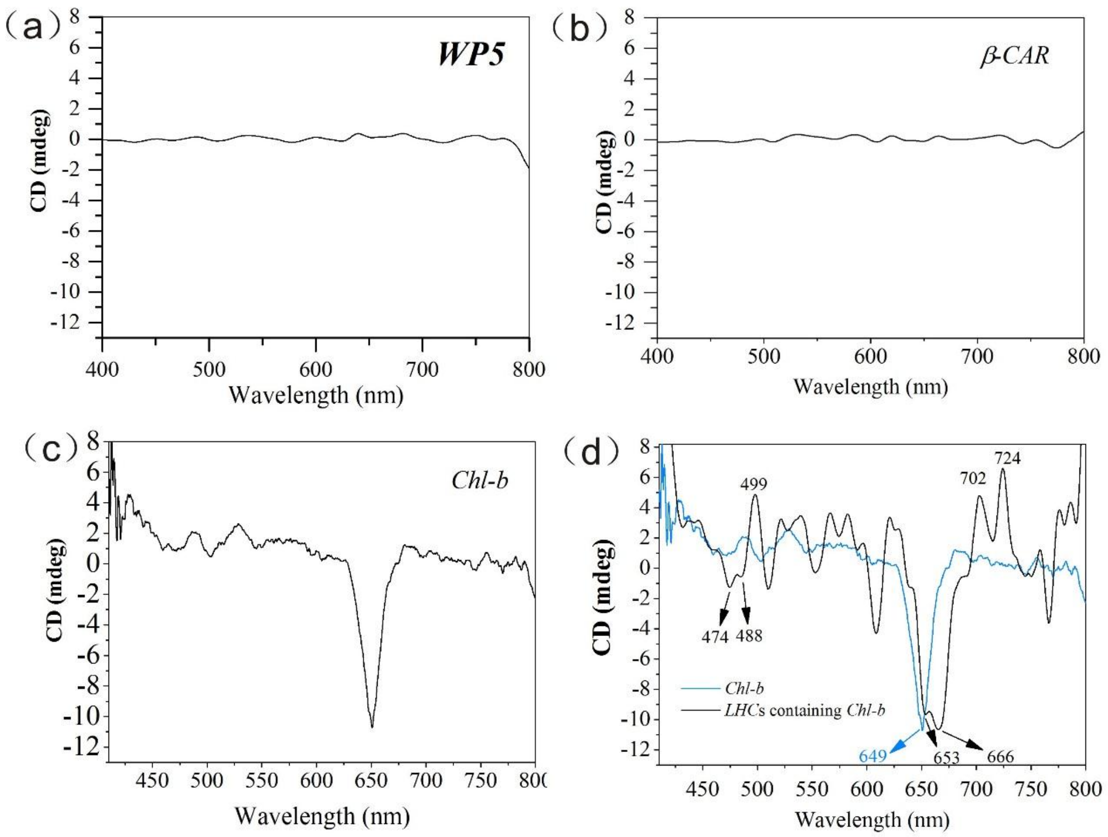

Supplementary Figure 20: CD spectra. CD spectra of (a) aqueous solution of ***WP5*** at a concentration of 75 μM, (b) ethanol solution of ***β-CAR*** at a concentration of 75 μM, (c) ethanol solution of ***Chl-b*** at a concentration of 75 μM, (d) normalized CD spectra of ***Chl-b*** and ***LHCs*** containing ***Chl-b*** (H_2O/ Ethanol =1:1).

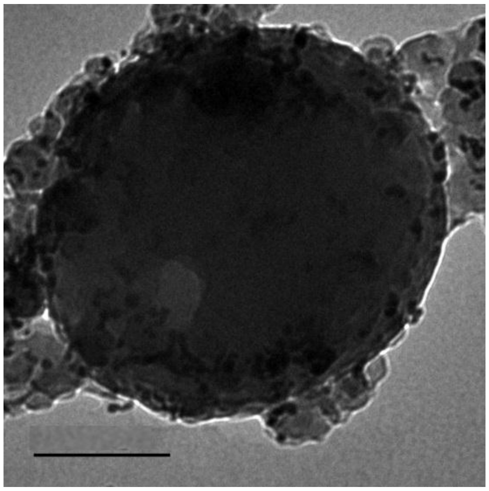

Supplementary Figure 21: TEM image of a AgNP-loaded *HMS*. Scale bar 200 nm.

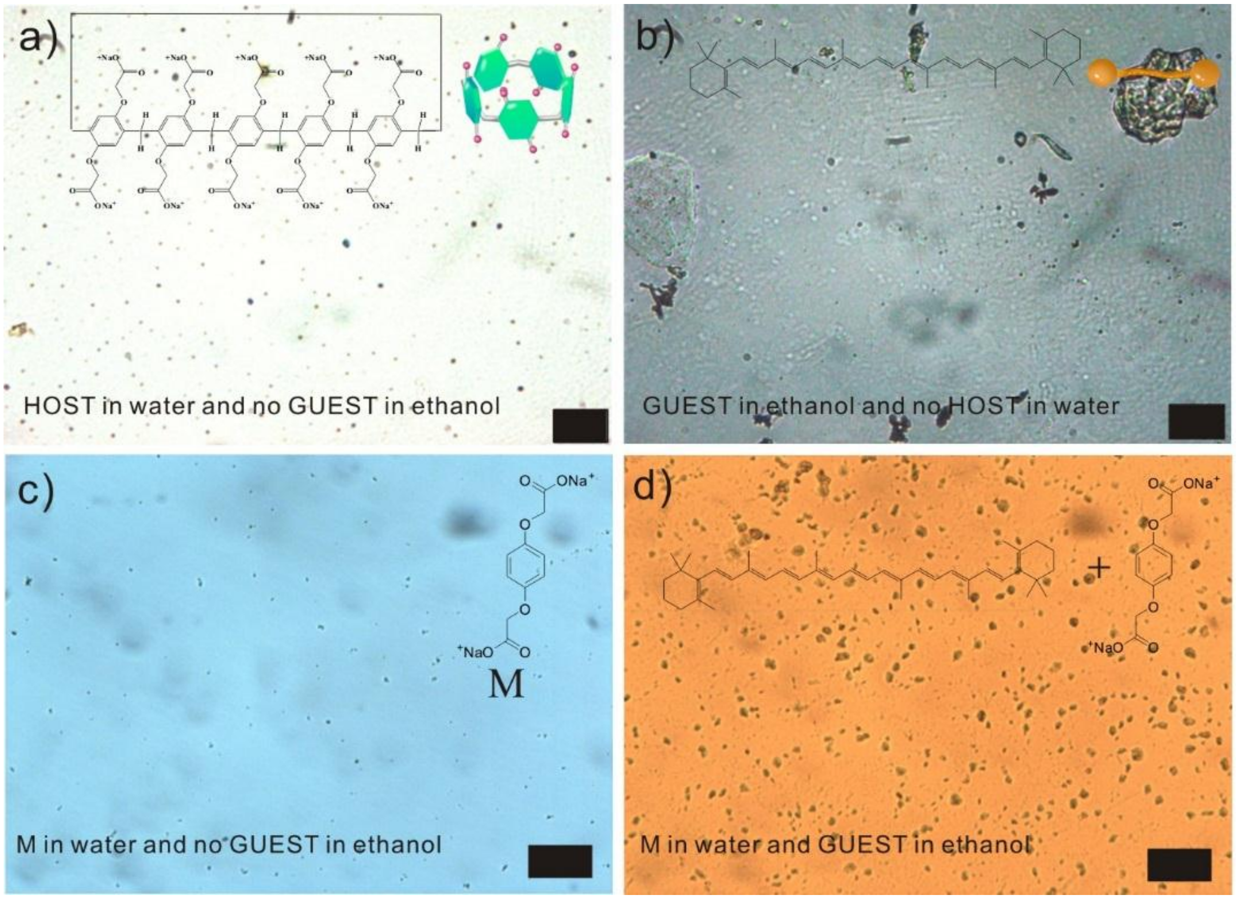

Supplementary Figure 22: Optical microscopy images. (a) replacing ***β-CAR*** from water, which does not undergo host-guest interactions and produced no ***HMSs***, scale bar, 10 μm, (b) replacing ***WP5*** from ethanol, which does not undergo host-guest interactions and produced no ***HMSs***, scale bar, 10 μm, (c) M in water and no ***β-CAR*** in ethanol, scale bar 20 μm. (d) M in water and ***β-CAR*** in ethanol, scale bar 20 μm.

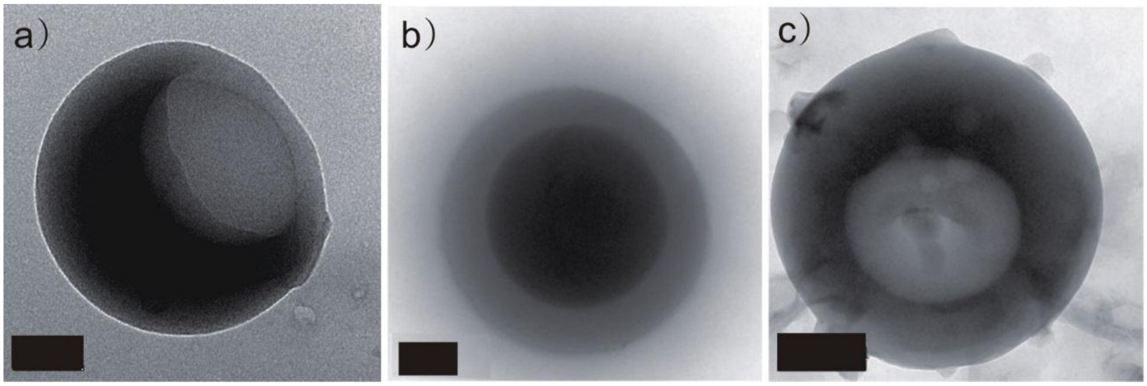

Supplementary Figure 23: TEM images demonstrating the stability of the *HMSs* to the external environment. No structural damage was observed after the addition of magnesium chloride (**a**), scale bar 50 nm, copper dichloride (**b**), scale bar 200 nm, and glutamic acid (**c**), scale bar 200 nm.

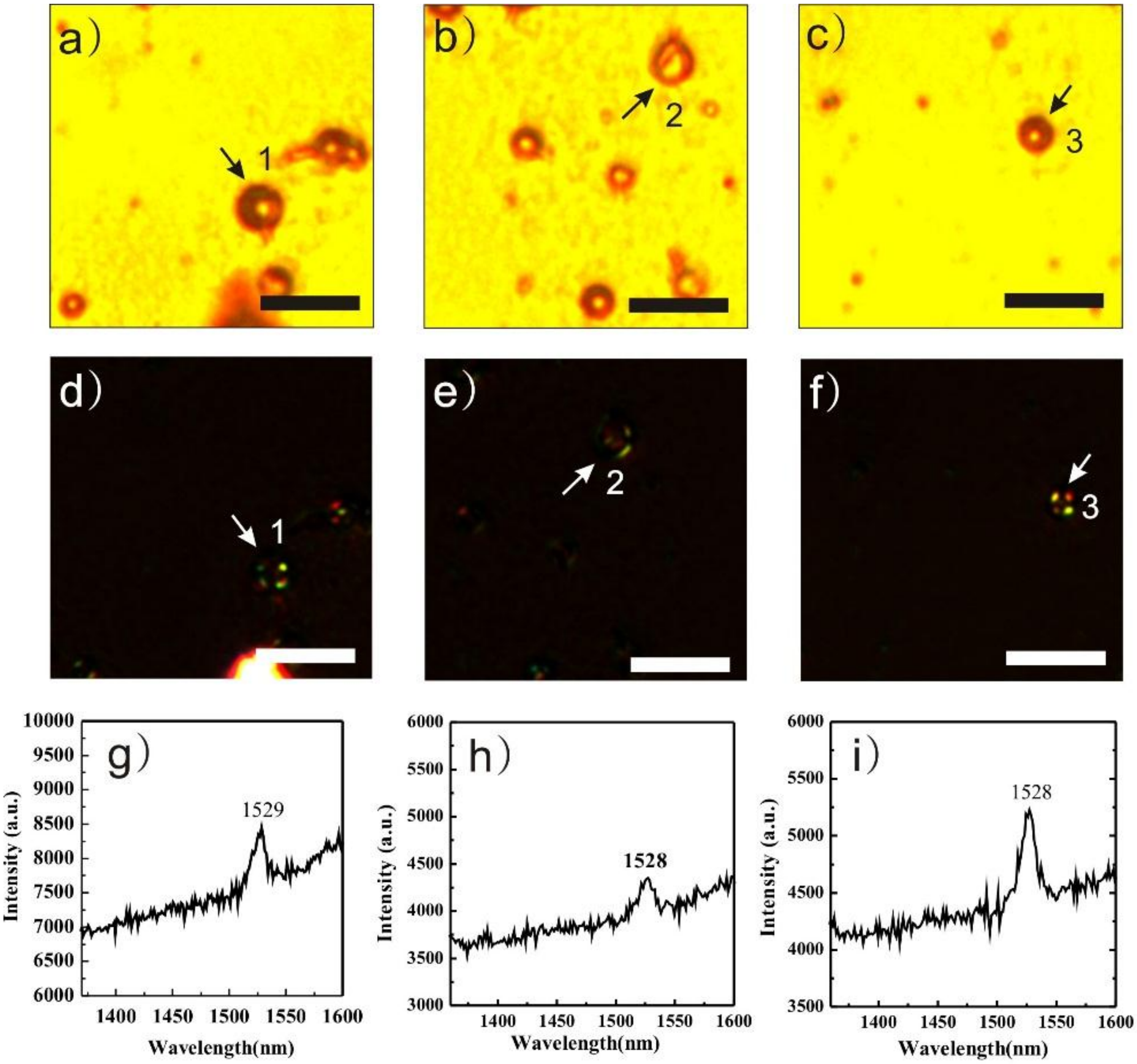

Supplementary Figure 24: Stability of *β-CAR* in *HMSs*. Individual *HMSs* were selected randomly from a sample and represented by 1, 2, and 3. (a)-(c) Optical microscopy images of ***WCC***-based ***HMSs*** dispersed on glass slides for more than six months. The hollow microspherical morphology demonstrates the stability of the ***HMSs*** at the supramolecular level. Scale bar 10 μm. **(d)-(f)** In situ polarized microscopy images of ***WCC***-based ***HMSs*** on glass slides after more than six months. The Maltese cross observed provides another important piece of evidence demonstrating the stability of the ***WP5*** and ***β-CAR*** building blocks based on ordered arrays. Scale bar 10 μm. **(g)-(i)** In situ Raman peaks at 1529 cm^{-1}, 1528 cm^{-1}, and 1528 cm^{-1} corresponding to the surface information about the ***HMSs*** in a, b, and c, suggesting that the stability to light and oxygen species increased when ***WP5*** protected the ***HMSs***.

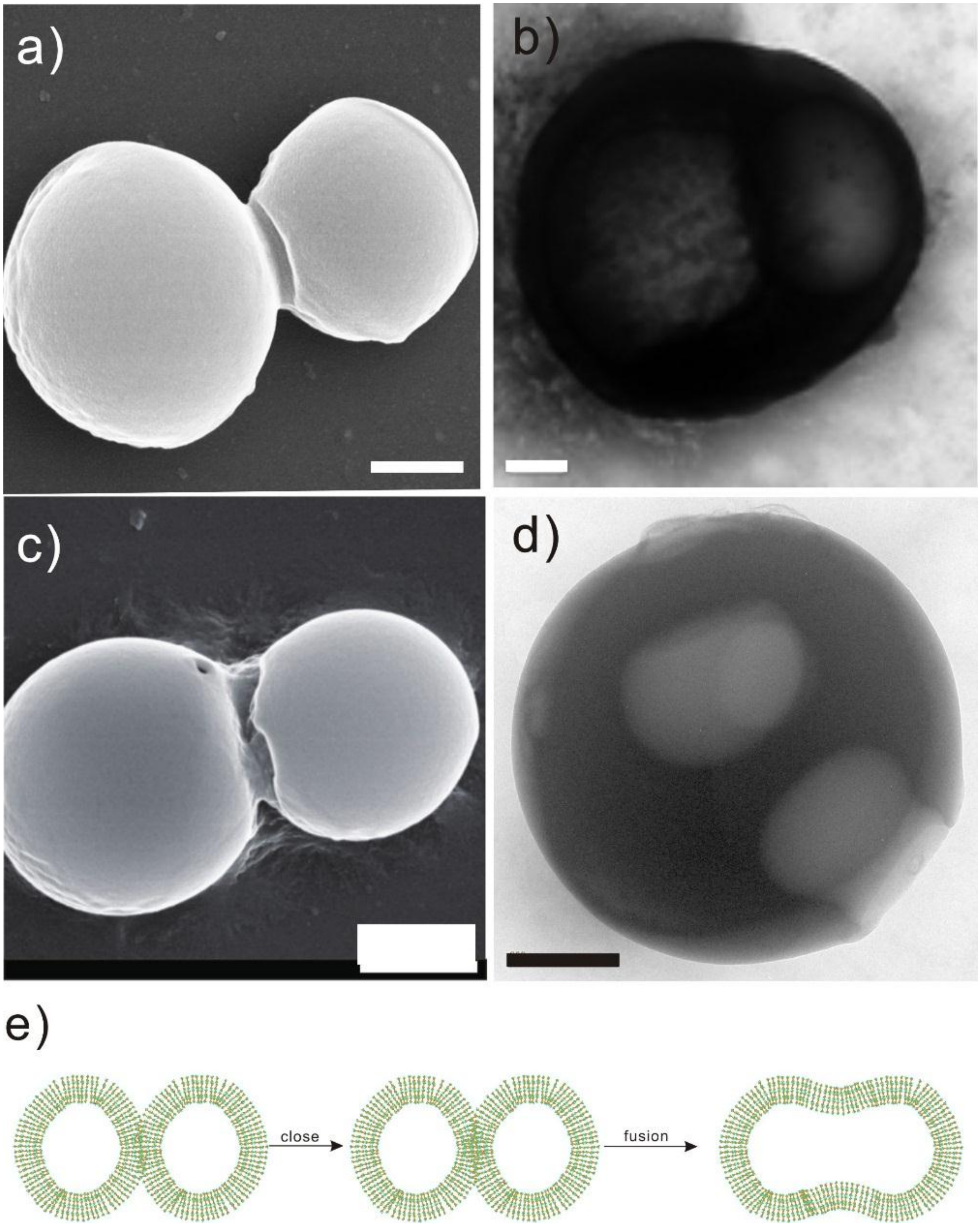

Supplementary Figure 25: *β-CAR* endows *HMSs* with special features. (a) SEM image of two fusing ***HMS***s. Scale bar, 500 nm. **(b)** TEM image showing a closed ***HMS*** possessing two chambers. Scale bar 200 nm. **(c)** SEM image of two fusing ***HMS***s. Scale bar 1 μm. **(d)** TEM image showing an ***HMS*** possessing two chambers with an open part. Scale bar 200 nm. **(e)** Cartoon of the fusion process for two ***HMS***s. For clarity, a bilayer is used in place of the multilayers to represent the evolution of two ***HMS***s.

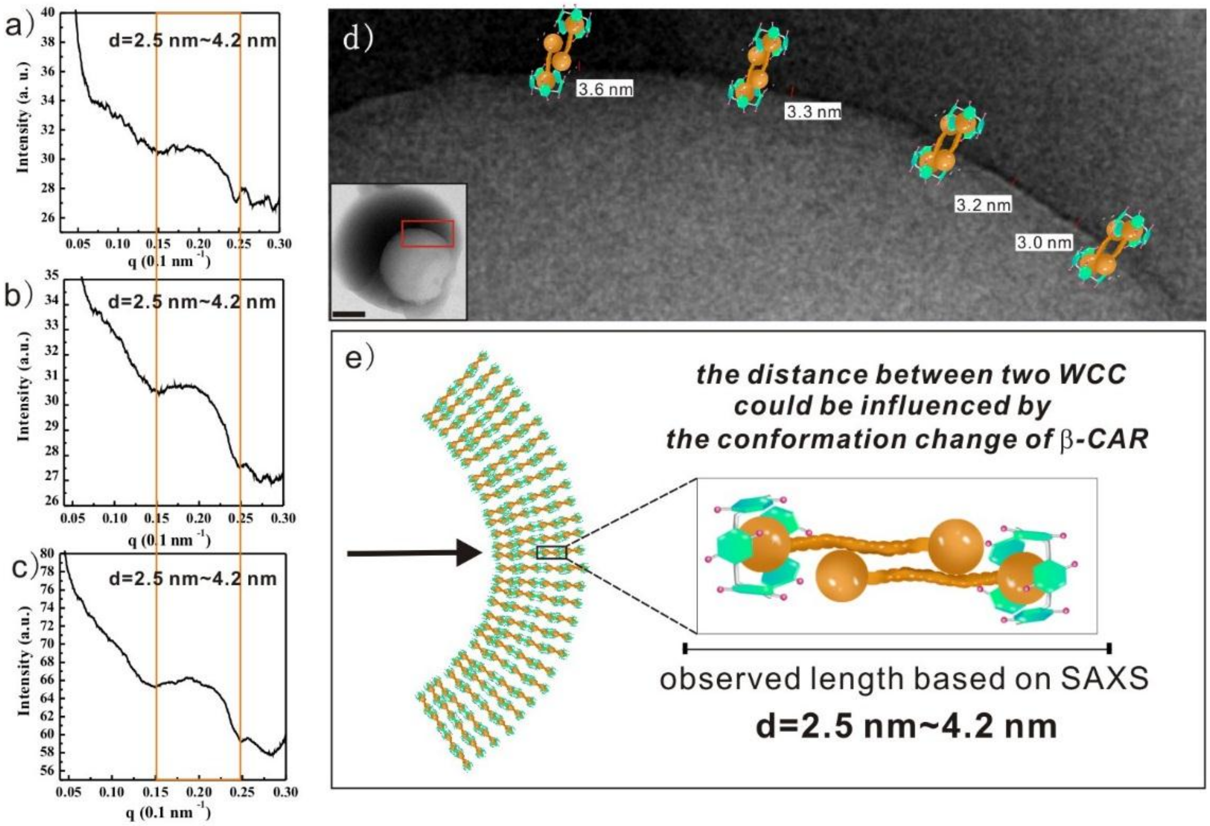

Supplementary Figure 26: The intensity of the peak increased with increasing acquisition time. SAXS profile of the ***HMS*** collected for different acquisition times (the final concentration of both the ***HMSs*** and ***β-CAR*** is 75 μM). (a) Profile of the ***HMSs*** collected at an acquisition time of 60 min. (b) Profile of the ***HMSs*** collected at an acquisition time of 120 min. (c) Profile of the ***HMS*** collected at an acquisition time of 240 min. All of these data exhibit broad peaks covering the *q* region range from 0.15 $Å^{-1}$ to 0.25 $Å^{-1}$, corresponding to an interlayer spacing between 2.5 nm and 4.2 nm. In addition, two small features at lower *q* values (at approximately 0.05 and 0.1 $Å^{-1}$) could be found in the profile, indicating that multiple structural orders with interlayer spacings between 2.5 nm and 4.2 nm exist in the ***HMSs***. (d) Cartoon representing the interlayer spacing centred at 3.0 nm, which might result from the conformational changes in the unsaturated carbon chain (the TEM image in d was only used to represent the difference in bilayer thickness for clarity). Scale bar 200 nm. (e) Cartoon of ***WCC***-based multilayers in ***HMSs***.

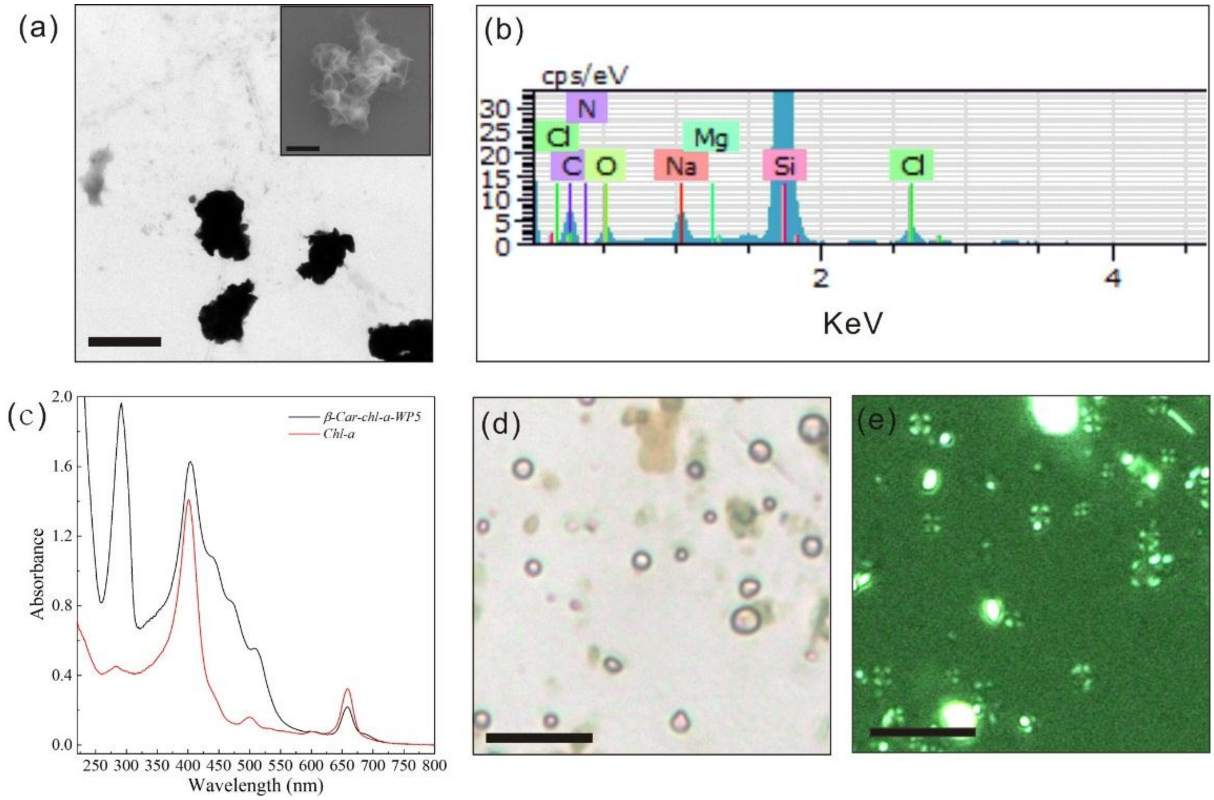

Supplementary Figure 27: *Chl-a* aggregates. (a) TEM image of ***Chl-a*** aggregates in absence of ***WP5*** and ***β-CAR*** (inset: corresponding SEM image. Scale bar 2 μm.). Scale bar 2 μm. (b) Energy spectrum of corresponding aggregates. (c) UV-vis spectra of ***Chl-a*** and ***LHCs*** containing ***Chl-a***. (d) Optical microscopy image showing ***LHCs*** containing ***Chl-a***. Scale bar 10 μm. (e) Polarized image of ***LHCs*** containing ***Chl-a*** (the colour in this image is false). Scale bar 10 μm.

Supplementary Discussion

Size matching plays an important role in ***WP5*** and ***β-CAR*** complexation. The molecular size in the energy minimization fits well within the cavity of ***WP5***. Considering the maximum length of the extended bulky terpene is slightly larger than the width of the cavity, ***β-CAR*** might exhibit the twisted conformation in the cavity. Importantly, during the construction process, the water/ethanol binary solvents increase the solubility of ***β-CAR***, which, in turn, facilitates the host-guest interaction between ***WP5*** and ***β-CAR***. The formation of the ***WP5⊃β-CAR*** complex is supported by the ^{1}H-NMR spectra, which show that the binding site was in the terpene section of ***β-CAR*** based on the hydrophobic interactions. In contrast to the chemical shift of the bonded terpene caused by solubilization and host-guest interactions, there was no obvious signal of unbounded terpene that might be caused by the obstruction of movement in the binary solvent. Thus, the stoichiometry should be determined through further characterization. The association constant of ***WP5*** and ***β-CAR*** was obtained using a non-linear curve-fitting method and fluorescence titration experiments. As shown in Supplementary Fig. 3g, upon addition of ***β-CAR***, the fluorescence intensity of ***WP5*** (monitored at 653 nm) was gradually quenched, and the association constant of ***WP5⊃β-CAR*** was calculated to be $(2.34 \pm 2.06) \times 10^{5}$ M^{-1} (Supplementary Fig. 3j).

As shown in Supplementary Fig. 11-13, the chemical shifts of protons H_a, H_b, H_c, H_d, and H_e of the alkyl chain protons on ***Chl-b*** shifted upfield slightly ($\Delta\delta(H_a)$= 0.040 ppm, $\Delta\delta(H_b)$= 0.048 ppm, $\Delta\delta(H_c)$= 0.051 ppm, $\Delta\delta(H_d)$= 0.020 ppm, and $\Delta\delta(H_e)$= 0.020 ppm). Furthermore, the peaks of the methyl protons H_f, H_g, H_h, H_j, and H_k on porphyrin shifted downfield slightly ($\Delta\delta(H_f)$= -0.018 ppm, $\Delta\delta(H_g)$= -0.021 ppm, $\Delta\delta(H_h)$= -0.043 ppm, $\Delta\delta(H_j)$= -0.010 ppm, and $\Delta\delta(H_k)$= -0.060 ppm). The methylene protons of the ethyl on the porphyrin were split into a doublet and triplet from a multiplet. However, this phenomenon does not affect the general trend of the interaction between ***WP5*** and ***Chl-b***, which suggested that a weak interaction exists in ***WP5⊃Chl-b*** (***WCB***). However, the obstruction of the movement of ***Chl-b*** caused by ***WP5*** is weaker than that of ***β-CAR*** in ***WP5⊃β-CAR*** complexation, which could also be demonstrated by the similar peak intensities before and after complexation.

Stable ***WP5*&*Chl-b*** complexation could not be achieved between ***WP5*** and ***Chl-b*** due to the size mismatch between the alkyl chain and the ***WP5*** cavity. Thus, ***β-CAR*** can insert into the unused cavity during the rapid movement of ***Chl-b*** into and out of the cavity. In this case, a large amount of ***WCC*** and free ***Chl-b*** exist for further assembly. Importantly, the hydrophobic tail of ***Chl-b*** (2.4 nm) fits well with the length of the hydrophobic moieties of ***WCC*** (2.3 nm), facilitating the location of ***Chl-b*** into the hydrophobic segment of the ***WCC*** through the hydrophobic tail and further promoting the coassembly between ***WCC*** and ***Chl-b*** on a supramolecular level. The association constant of ***WP5*** and ***Chl-b*** was obtained using a non-linear curve-fitting method and fluorescence titration experiments. According to the different emission peaks of ***WP5*** and ***Chl-b*** in the range from 300 to 750 nm, the peak at 450 nm was selected to monitor the intensity change of ***Chl-b*** (Supplementary Fig. 14a-b). As shown in Supplementary Fig. 14c-d, according to the fluorescence intensity change of ***Chl-b*** (monitored at 450 nm), the association constant of ***WP5⊃Chl-b*** was calculated to be $(3.46 \pm 0.14) \times 10^3$ M^{-1} (Supplementary Fig. 14e).

Based on the optical microscopy observation (Supplementary Fig. 17), it was found that ***LHCs-b*** with diameters larger than 2 μm possess deep-orange and green walls (red square), indicating the co-existence of ***β-CAR*** and ***Chl-b*** in the walls. For the ***LHCs-b*** with diameters less than 650 nm (blue squares), it is difficult to distinguish the hollow structure and fine compositions of the walls by optical microscopy. Thus, TEM and EDX mapping observations were used for further characterization. The fine element distributions in ***LHCs-b*** were further characterized by EDX mapping. As shown in **S**upplementary Fig. 18**,** the N and Mg distributions in ***LHCs-b*** with different diameters further demonstrated that ***Chl-b*** successfully participated in the construction of ***LHCs-b***. As shown in Supplementary Fig. 18 a, ***LHC-b*** with diameters of 200 nm were observed, which not only showed that ***Chl-b*** could participate in the construction of small sphere walls but also indicated that the insertion of ***Chl-b*** did not destroy the hierarchical structure. Supplementary Fig. 18b shows the EDX mapping, which further demonstrated the existence of ***Chl-b*** in ***HMS*** with diameters of 400 nm. Supplementary Fig. 18c shows the EDX mapping of an ***LHC-b*** with a diameter of 900 nm, which further demonstrated the existence of ***Chl-b*** in hollow spheres with larger diameters; the black region in the image was caused by damage to the internal structure of hollow sphere. Supplementary Fig. 18d shows the EDX mapping of an ***LHC-b*** with a diameter of 1000 nm, which not only demonstrated the existence of ***Chl-b*** in ***HMS*** with larger diameters but also demonstrated the more homogeneous wall thickness. Interestingly, for the ***LHC-b*** with

diameters larger than 1500 nm (Supplementary Fig. 18e), a fusion phenomenon was observed, which provided the possibility of integration and growth.

In order to calculate the ***β-CAR***/***Chl-b*** ratio in a single ***LHC-b*** and further analyse these data, a series of ***LHC***s-***b*** were selected randomly. Subsequently, the atomic percentage of ***Na*** (1 ***β-CAR*** ~ 1 ***WP5*** ~ 10 ***Na***) and ***Mg*** (1 ***Chl-b*** ~ 1 ***Mg***) in ***LHC***s-***b*** was chosen to be investigated (Supplementary Fig. 19). It should be noted that the light elements[3] C, N, and O with an atomic number less than 10 result in their peaks areas overlapping with each other at a large scale, which makes it difficult to distinguish the characteristic peaks. Thus, Na and Mg (with atomic numbers more than 10) were selected as the characteristic elements of ***WP5*** and ***Chl-b***, respectively, for calculating the ratio of ***WP5 (β-CAR)*** to ***Chl-b***. ***LHC***s-***b*** with different ***β-CAR***/***Chl-b*** ratios (2.0, 2.4, 1.8, and 1.0) were obtained by calculation, demonstrating the random manner of ***Chl-b*** in ***LHC***s-***b***. Based on the different content of ***Na*** and ***Mg*** in individual ***LHC***s-***b***, the average ratio of 1.8 was obtained by further calculation. However, these data are provided only for providing a rough estimate. Some factors, such as the limitation of EDX spectra accuracy and ***Chl-b*** inside the spheres, make the results exhibit a large deviation; thus, these semi-quantitative analysis data are provided for reference only.

The generation of an ***HMS*** must be associated with the structural characteristics of the ***WCC***; when either ***WP5*** or ***β-CAR*** was removed from the water or ethanol, no ***HMS*** formation was observed (Supplementary Fig. 22 a-b). In addition, a model compound M (Supplementary Fig. 22 c) was used to prove the importance of the host–guest interactions in ***HMS*** construction; however, no notable hierarchical nanostructure (Supplementary Fig. 22 d) was observed when the ***β-CAR*** was added to the solution of M. These phenomena indicated that the host−guest-based ***WCC*** complexation plays an essential role in the formation of ***HMS***, mainly driven by hydrophobic interactions and hydrogen bonds. The above results demonstrate that hydrogen bonding (CH ····O), CH ····π interactions, hydrophobic interactions, and π−π stacking interactions reinforce the complex stability.

An ***HMS*** exhibits stability against the external environment. No structural damage was observed after the addition of magnesium chloride, copper dichloride, and glutamic acid (Supplementary Fig. 23). An ***HMS*** exhibits stability over time. Supplementary Fig. 24 a-c shows optical microscopy images of ***WCC***-based ***HMSs*** on glass slides over the course of more than six months. The preservation of the hollow microspherical morphology demonstrates the stability of

the ***HMS***s on the supramolecular level. In addition, the stability beyond the molecular level of ***HMS***s is also supported by in situ polarized microscopy observations. The Maltese cross observed in Supplementary Fig. 24 d-f provides another important piece of evidence for the existence of ***WP5*** and ***β-CAR*** building blocks based on ordered arrays. More importantly, the ***HMS*** wall remains orange in colour, implying the stability of ***β-CAR*** on the molecular level. The in situ Raman peaks at 1529 cm^{-1}, 1528 cm^{-1}, and 1528 cm^{-1}, which correspond to the surface information regarding the ***HMS***s in a, b, and c, suggest that the stability to light and oxygen species was increased in favour of the protection of ***WP5*** in the ***HMS*** (Supplementary Fig. 24 g-i).

The ***HMS*** were observed to display characteristics that differ from those of traditional synthetic molecule-based suprastructures. As shown in Supplementary Fig. 25, two small spheres can attach to each other spontaneously, resulting in what could be considered the intermediate of the fusion process. The interactions between two ***HMS***s might be ascribed to the conformational change of the unsaturated carbon chain in ***β-CAR***, which leads to the fluidity between the layers and further promotes the growth of microspheres. Furthermore, the peak in the SAXS profile is weak as a consequence of the low concentration of ***HMS***s (the final concentration of ***WP5*** and ***β-CAR*** is 75 μM) and the power limitation of the SAXS instrument (30 W). Thus, to overcome these disadvantages and further obtain convincing data, the acquisition time for detection was increased. As shown in Supplementary Fig. 26, the intensity of the peak increased with increasing acquisition time.

The purpose of our work is to provide a platform for the construction of diverse artificial biological cells. Thus, ***Chl-a*** was introduced to verify the feasibility of the proposed method. As shown in Supplementary Fig. 27, ***LHC*** containing ***Chl-a*** could be obtained based on ***WCC***-based ***HMS***.

Supplementary References

1. Doering, W., Sotiriou-Leventis, C. & Roth, W. Thermal Interconversion among 15-cis, 13-cis and all-trans-β-Carotene: Kinetics, Arrhenius Parameters, Thermochemistry and Potential Relevance to Anticarcinogenicity of all-trans- β-Carotene. *J. Am. Chem. Soc*. **117**, 2747-2757 (1995).

2. Wu, S. & Rebeiz, C. Chloroplast Biogenesis Molecular Structure of Chlorophyll b. *J. Biol. Chem*. **260**, 3632-3634 (1985).
3. Ohfuji, H. & Yamamoto. M. EDS quantification of light elements using osmium surface coating. *J. Miner. Petrol. Sci*. **110**, 189-195 (2015).
